# Supplementary material for: Bayesian inference of relative fitness on high-throughput pooled competition assays
Source: PLoS Comput Biol. 2024 Mar 15;20(3):e1011937. doi: 10.1371/journal.pcbi.1011937 (PMC10971673; doi:10.1371/journal.pcbi.1011937)
Supplement: S1 Text — Section A gives a short primer on variational inference. Section B defines the probabilistic models used throughout the main text. Section C details how the validity of the model is computed via posterior predictive checks. Section D explains how the simulated frequency trajectories are generated. Section E compares the inferences of our method with state-of-the-art methods in the literature. Section F reanalyzes experimental data from yeast evolution experiments. Section G details how the computation time scales with the number of barcodes. (PDF) [file pcbi.1011937.s001.pdf]

Table of contents

Supplementary Materials **1**

A. Primer on Variational Inference . . . 1

A.1. ADVI algorithm . . . 5

B. Defining the Bayesian model . . . 6

B.1. Frequency uncertainty $\pi(\underline{\underline{F}} \mid \underline{\underline{R}})$ . . . 7

B.2. Population mean fitness uncertainty $\pi(\underline{\bar{s}}_T \mid \underline{\underline{F}}, \underline{\underline{R}})$ . . . 9

B.3. Mutant relative fitness uncertainty $\pi(\underline{s}^M \mid \underline{\bar{s}}_T, \underline{\underline{F}}, \underline{\underline{R}})$ . . . 12

B.4. Hierarchical models for multiple experimental replicates . . . 15

B.5. Defining prior probabilities . . . 18

C. Posterior predictive checks . . . 19

D. Logistic growth simulation . . . 21

D.1. Simulation details . . . 23

E. Comparison with `FitSeq2.0` . . . 28

F. Reanalyzing Kinsler et al., 2020 . . . 31

F.1. Analysis of individual replicates . . . 31

F.2. Hierarchical analysis of multiple replicates . . . 34

G. Computation time scaling . . . 41

Supplementary Materials

A. Primer on Variational Inference

In this section, we will briefly introduce the idea behind variational inference. Recall that any Bayesian inference problem deals with the joint distribution between observations $\underline{x}$ and unobserved latent variables $\underline{\theta}$. This joint distribution can be written as the product of a distribution of the observations $\underline{x}$ conditioned on the $\underline{\theta}$ and the marginal distribution of these latent variables, i.e.,

$$\pi(\underline{x}, \underline{\theta}) = \pi(\underline{x} \mid \underline{\theta})\pi(\underline{\theta}). \tag{S1}$$

A Bayesian inference pipeline's objective is to compute the latent variables' posterior probability given a set of observations. This computation is equivalent to updating our prior beliefs about the set of values that the latent variables take after taking in new data. We write this as Bayes theorem

$$\pi(\underline{\theta} \mid \underline{x}) = \frac{\pi(\underline{x} \mid \underline{\theta})\pi(\underline{\theta})}{\pi(\underline{x})}. \tag{S2}$$

The main technical challenge for working with Equation S2 comes from the computation of the denominator, also known as the *evidence* or the *marginalized likelihood*. The reason

computing this term is challenging is because it involves a (potentially) high-dimensional integral of the form

$$\pi(\underline{x}) = \int \cdots \int d^K \underline{\theta}\, \pi(\underline{x}, \underline{\theta}) = \int \cdots \int d^K \underline{\theta}\, \pi(\underline{x} \mid \underline{\theta}) \pi(\underline{\theta}), \tag{S3}$$

where K is the dimesionality of the $\underline{\theta}$ vector. Here, the integrals are taken over the support—the set of values valid for the distribution—of $\pi(\underline{\theta})$. However, only a few selected distributions have a closed analytical form; thus, in most cases Equation S3 must be solved numerically.

Integration in high-dimensional spaces can be computationally extremely challenging. For a naive numerical quadrature procedure, integrating over a grid of values for each dimension of $\underline{\theta}$ comes with an exponential explosion of the number of required grid point evaluations, most of which do not contribute significantly to the integration. To gain visual intuition about this challenge, imagine integrating the function depicted in Figure A. If the location of the high-density region (dark peak) is unknown, numerical quadrature requires many grid points to ensure we capture this peak. However, most of the numerical evaluations of the function on the grid points do not contribute significantly to the integral. Therefore, our computational resources are wasted on insignificant evaluations. This only gets worse as the number of dimensions increases since the number of grid point evaluation scales exponentially.

Modern Markov Chain Monte Carlo algorithms, such as Hamiltonian Monte Carlo, can efficiently perform this high-dimensional integration by utilizing gradient information from the target density Betancourt [1]. Nevertheless, these sampling-based methods become prohibitively slow for the number of dimensions our present inference problem presents. Thus, there is a need to find scalable methods for the inference problem in Equation S2.

Variational inference circumvents these technical challenges by proposing an approximate solution to the problem. Instead of working with the posterior distribution in its full glory $\pi(\underline{\theta} \mid \underline{x})$, let us propose an approximate posterior distribution q_ϕ that belongs to a distribution family fully parametrized by ϕ. For example, let us say that the distribution q_ϕ belongs to the family of multivariate Normal distributions such that $\phi = (\underline{\mu}, \underline{\underline{\Sigma}})$, where $\underline{\mu}$ is the vector of means and $\underline{\underline{\Sigma}}$ is the covariance matrix. If we replace π by q_ϕ, we want q_ϕ to resemble the original posterior as much as possible. Mathematically, this can be expressed as minimizing a "*distance metric*"—the Kullback-Leibler (KL) divergence, for example—between the distributions. Note that we use quotation marks because, formally, the KL divergence is not a distance metric since it is not symmetric. Nevertheless, the variational objective is set to find a distribution q^*_ϕ such that

$$q^*_\phi(\underline{\theta}) = \min_\phi D_{KL}\left(q_\phi(\underline{\theta}) || \pi(\underline{\theta} \mid \underline{x})\right), \tag{S4}$$

where D_{KL} is the KL divergence. Furthermore, we highlight that the KL divergence is a strictly positive number, i.e.,

$$D_{KL}\left(q_\phi(\underline{\theta}) || \pi(\underline{\theta} \mid \underline{x})\right) \geq 0, \tag{S5}$$

as this property will become important later on.

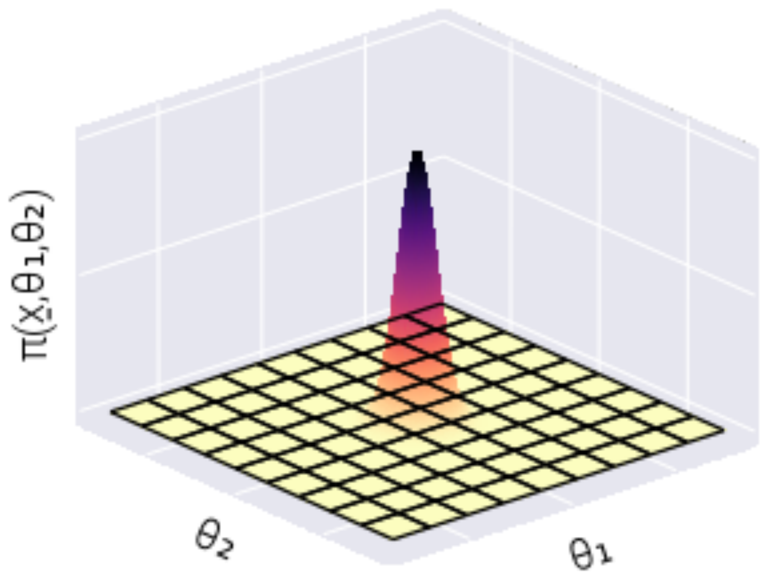

Figure A. High-dimensional numerical quadrature does not scale with dimensionality. Schematic depiction of the problem with naive numerical quadrature to integrate over an unknown density. While the density is concentrated on the dark peak, most of the evaluations over the $x_1 - x_2$ grid do not contribute to the value of the integral

At first sight, Equation S4 does not improve the situation but only introduces further technical complications. After all, the definition of the KL divergence

$$D_{KL}\left(q_\phi(\underline{\theta})||\pi(\underline{\theta} \mid \underline{x})\right) \equiv \int \cdots \int d^K\underline{\theta}\; q_\phi(\underline{\theta}) \ln \frac{q_\phi(\underline{\theta})}{\pi(\underline{\theta} \mid \underline{x})}, \tag{S6}$$

includes the posterior distribution $\pi(\underline{\theta} \mid \underline{x})$ we are trying to get around. However, let us manipulate Equation S6 to beat it to a more reasonable form. First, we can use the properties of the logarithms to write

$$D_{KL}\left(q_\phi(\underline{\theta})||\pi(\underline{\theta} \mid \underline{x})\right) = \int d^K\underline{\theta}\; q_\phi(\underline{\theta}) \ln q_\phi(\underline{\theta}) - \int d^K\underline{\theta}\; q_\phi(\underline{\theta}) \ln \pi(\underline{\theta} \mid \underline{x}), \tag{S7}$$

where, for convenience, we write a single integration sign ($d^K\underline{\theta}$ still represents a multi-dimensional differential). For the second term in Equation S7, we can substitute the term inside the logarithm using Equation S2. This results in

$$\begin{aligned} D_{KL}\left(q_\phi(\underline{\theta})||\pi(\underline{\theta} \mid \underline{x})\right) = &\int d^K\underline{\theta}\; q_\phi(\underline{\theta}) \ln q_\phi(\underline{\theta}) \\ &- \int d^K\underline{\theta}\; q_\phi(\underline{\theta}) \ln \left(\frac{\pi(\underline{x} \mid \underline{\theta})\pi(\underline{\theta})}{\pi(\underline{x})}\right). \end{aligned} \tag{S8}$$

Again, using the properties of logarithms, we can split Equation S8, obtaining

$$\begin{aligned} D_{KL}\left(q_\phi(\underline{\theta})||\pi(\underline{\theta} \mid \underline{x})\right) = &\int d^K\underline{\theta}\; q_\phi(\underline{\theta}) \ln q_\phi(\underline{\theta}) \\ &- \int d^K\underline{\theta}\; q_\phi(\underline{\theta}) \ln \pi(\underline{x} \mid \underline{\theta}) \\ &- \int d^K\underline{\theta}\; q_\phi(\underline{\theta}) \ln \pi(\underline{\theta}) \\ &+ \int d^K\underline{\theta}\; q_\phi(\underline{\theta}) \ln \pi(\underline{x}). \end{aligned} \tag{S9}$$

It is convenient to write Equation S9 as

$$\begin{aligned} D_{KL}\left(q_\phi(\underline{\theta})||\pi(\underline{\theta} \mid \underline{x})\right) = &\int d^K\underline{\theta}\; q_\phi(\underline{\theta}) \ln \frac{q_\phi(\underline{\theta})}{\pi(\underline{\theta})} \\ &- \int d^K\underline{\theta}\; q_\phi(\underline{\theta}) \ln \pi(\underline{x} \mid \underline{\theta}) \\ &+ \ln \pi(\underline{x}) \int d^K\underline{\theta}\; q_\phi(\underline{\theta}), \end{aligned} \tag{S10}$$

where for the last term, we can take $\ln \pi(\underline{x})$ out of the integral since it does not depend on $\underline{\theta}$. Lastly, we utilize two properties:

1. The proposed approximate distribution must be normalized, i.e.,

$$\int d^K\underline{\theta}\; q_\phi(\underline{\theta}) = 1. \tag{S11}$$

2. The law of the unconscious statistician (LOTUS) establishes that for any probability density function, it must be true that

$$\int d^K \underline{\theta}\, q_\phi(\underline{\theta}) f(\underline{\theta}) = \langle f(\underline{\theta}) \rangle_{q_\phi}, \tag{S12}$$

where $\langle \cdot \rangle_{q_\phi}$ is the expected value over the q_ϕ distribution.

Using these two properties, the positivity constraint on the KL divergence in Equation S5, and the definition of the KL divergence in Equation S6 we can rewrite Equation S10 as

$$D_{KL}\left(q_\phi(\underline{\theta}) || \pi(\underline{\theta})\right) - \langle \ln \pi(\underline{x} \mid \underline{\theta}) \rangle_{q_\phi} \geq - \ln \pi(\underline{x}). \tag{S13}$$

Multiplying by a minus one, we have the functional form of the so-called evidence lower bound (ELBO) Kingma and Welling [2],

$$\underbrace{\ln \pi(\underline{x})}_{\text{log evidence}} \geq \underbrace{\langle \ln \pi(\underline{x} \mid \underline{\theta}) \rangle_{q_\phi} - D_{KL}\left(q_\phi(\underline{\theta}) || \pi(\underline{\theta})\right)}_{\text{ELBO}}. \tag{S14}$$

Let us recapitulate where we are. We started by presenting the challenge of working with Bayes' theorem, as it requires a high-dimensional integral of the form in Equation S3. As an alternative, variational inference posits to approximate the posterior distribution $\pi(\underline{\theta} \mid \underline{x})$ with a parametric distribution $q_\phi(\underline{\theta})$. By minimizing the KL divergence between these distributions, we arrive at the result in Equation S14, where the left-hand side—the log marginalized likelihood or log evidence—we cannot compute for technical/computational reasons. However, the right-hand side is composed of things we can easily evaluate. We can easily evaluate the log-likelihood $\ln \pi(\underline{x} \mid \underline{\theta})$ and the KL divergence between our proposed approximate distribution $q_\phi(\underline{\theta})$ and the prior distribution $\pi(\underline{\theta})$. Moreover, we can compute the gradients of these functions with respect to the parameters of our proposed distribution. This last point implies that we can change the parameters of the proposed distribution to maximize the ELBO. And, although we cannot compute the left-hand side of Equation S14, we know that however large we make the ELBO, it will always be smaller than (or equal) the log-marginal likelihood. Therefore, the larger we can make the ELBO by modifying the parameters ϕ, the closer it gets to the log-marginal likelihood, and, as a consequence, the better our proposed distribution $q_\phi(\underline{\theta})$ gets to the true posterior distribution $\pi(\underline{\theta} \mid \underline{x})$.

In this sense, variational inference turns the intractable numerical integration problem to an optimization routine, for which there are several algorithms available.

A.1. ADVI algorithm

To maximize the right-hand side of Equation S14, the Automatic Differentiation Variational Inference (ADVI) algorithm developed in [3] takes advantage of advances in probabilistic programming languages to generate a robust method to perform this optimization. Without

going into the details of the algorithm implementation, for our purposes, it suffices to say that we define our joint distribution $\pi(\underline{\theta}, \underline{x})$ as the product defined in Equation S1. ADVI then proposes an approximate variational distribution q_ϕ that can either be a multivariate Normal distribution with a diagonal covariance matrix, i.e.,

$$\phi = (\underline{\mu}, \underline{\underline{D}}), \tag{S15}$$

where $\underline{\underline{D}}$ is the identity matrix, with the diagonal elements given by the vector of variances $\underline{\sigma}^2$ for each variable or a full-rank multivariate Normal distribution

$$\phi = (\underline{\mu}, \underline{\underline{\Sigma}}). \tag{S16}$$

Then, the parameters are initialized in some value ϕ_o. These parameters are iteratively updated by computing the gradient of the ELBO (right-hand side of Equation S14), hereafter defined as $\mathcal{L}$, with respect to the parameters,

$$\nabla_\phi \mathcal{L} = \nabla_{\underline{\mu}} \mathcal{L} + \nabla_{\underline{\sigma}} \mathcal{L}, \tag{S17}$$

and then computing

$$\phi_{t+1} = \phi_t + \eta \nabla_\phi \mathcal{L},$$

where η defines the step size.

This short explanation behind the ADVI algorithm is intended only to gain intuition on how the optimal variational distribution q_ϕ be computed. There are many nuances in the implementation of the ADVI algorithm. We invite the reader to look at the original reference for further details.

B. Defining the Bayesian model

In the main text, we specify the inference problem we must solve as being of the form

$$\pi(\underline{s}^M, \underline{\bar{s}}_T, \underline{\underline{F}} \mid \underline{\underline{R}}) \propto \pi(\underline{\underline{R}} \mid \underline{s}^M, \underline{\bar{s}}_T, \underline{\underline{F}}) \pi(\underline{s}^M, \underline{\bar{s}}_T, \underline{\underline{F}}). \tag{S18}$$

Here, we briefly define the missing nuisance parameters. Let

$$\underline{\bar{s}}_T = (\bar{s}_1, \bar{s}_2, \ldots, \bar{s}_{T-1})^\dagger, \tag{S19}$$

be the vector containing the $T-1$ population mean fitness we compute from the T time points where measurements were taken. We have $T-1$ since the value of any $\bar{s}_t$ requires cycle numbers t and $t+1$. Furthermore, let the matrix $\underline{\underline{F}}$ be a $T \times B$ matrix containing all frequency values. As with Equation 12 in the main text, we can split $\underline{\underline{F}}$ into two matrices of the form

$$\underline{\underline{F}} = \left[\underline{\underline{F}}^N \; \underline{\underline{F}}^M \right], \tag{S20}$$

to separate the corresponding neutral and non-neutral barcode frequencies.

Let us now define each of the terms in Equation 18 described in Section 2.4 of the main text. The following sections will specify the functional form each of these terms takes.

B.1. Frequency uncertainty $\pi(\underline{\underline{F}} \mid \underline{\underline{R}})$

We begin with the probability of the frequency values given the raw barcode reads. The first assumption is that the inference of the frequency values for time t is independent of any other time. Therefore, we can write the joint probability distribution as a product of independent distributions of the form

$$\pi(\underline{\underline{F}} \mid \underline{\underline{R}}) = \prod_{t=1}^{T} \pi(\underline{f}_t \mid \underline{r}_t), \tag{S21}$$

where $\underline{f}_t$ and $\underline{r}_t$ are the t-th row of the matrix containing all of the measurements for time t. We imagine that when the barcode reads are obtained via sequencing, the quantified number of reads is a Poisson sample from the "true" underlying number of barcodes within the pool. This translates to assuming that the number of reads for each barcode at any time point $r_t^{(b)}$ is an independent Poisson random variable, i.e.,

$$r_t^{(b)} \sim \text{Poiss}(\lambda_t^{(b)}), \tag{S22}$$

where the symbol "$\sim$" is read "distributed as." Furthermore, for a Poisson distribution, we have that

$$\lambda_t^{(b)} = \left\langle r_t^{(b)} \right\rangle = \left\langle \left(r_t^{(b)} - \left\langle r_t^{(b)} \right\rangle \right)^2 \right\rangle, \tag{S23}$$

where $\langle \cdot \rangle$ is the expected value. In other words the Poisson parameter is equal to the mean and variance of the distribution. The Poisson distribution has the convenient property that for two Poisson distributed random variables $X \sim \text{Poiss}(\lambda_x)$ and $Y \sim \text{Poiss}(\lambda_y)$, we have that

$$Z \equiv X + Y \sim \text{Poiss}(\lambda_x + \lambda_y). \tag{S24}$$

This additivity allows us to write the total number of reads at time t n_t also as a Poisson-distributed random variable of the form

$$n_t \sim \text{Poiss}\left(\sum_{b=1}^{B} \lambda_t^{(b)} \right), \tag{S25}$$

where the sum is taken over all B barcodes.

If the total number of reads is given by Equation S25, the array with the number of reads for each barcode at time t, $\underline{r}_t$ is then distributed as

$$\underline{r}_t \sim \text{Multinomial}(n_t, \underline{f}_t), \tag{S26}$$

where each of the B entries of the frequency vector $\underline{f}_t$ is a function of the $\underline{\lambda}_t$ vector, given by

$$f_t^{(b)} \equiv f_t^{(b)}(\underline{\lambda}_t) = \frac{\lambda_t^{(b)}}{\sum_{b'=1}^{B} \lambda_t^{(b')}}. \tag{S27}$$

In other words, we can think of the B barcode counts as independent Poisson samples or as a single multinomial draw with a random number of total draws, n_t, and the frequency vector $\underline{f}_t$ we are interested in. Notice that Equation S27 is a deterministic function that connects the Poisson parameters to the frequencies. Therefore, we have the equivalence that

$$\pi(\underline{f}_t \mid \underline{r}_t) = \pi(\underline{\lambda}_t \mid \underline{r}_t), \tag{S28}$$

meaning that the uncertainty comes from the $\underline{\lambda}_t$ vector. By Bayes theorem, we therefore write

$$\pi(\underline{\lambda}_t \mid n_t, \underline{r}_t) \propto \pi(n_t, \underline{r}_t \mid \underline{\lambda}_t)\pi(\underline{\lambda}_t), \tag{S29}$$

where we explicitly include the dependence on n_t. This does not affect the distribution or brings more uncertainty because $\underline{r}_t$ already contains all the information to compute n_t since

$$n_t = \sum_{b=1}^{B} r_t^{(b)}. \tag{S30}$$

But adding the variable allows us to factorize Equation S29 as

$$\pi(\underline{\lambda}_t \mid n_t, \underline{r}_t) \propto \pi(\underline{r}_t \mid n_t, \underline{\lambda}_t)\pi(n_t \mid \underline{\lambda}_t)\pi(\underline{\lambda}_t) \tag{S31}$$

We then have

$$\underline{r}_t \mid n_t, \underline{\lambda}_t \sim \text{Multinomial}(n_t, \underline{f}_t(\underline{\lambda}_t)). \tag{S32}$$

Furthermore, we have

$$n_t \mid \underline{\lambda}_t \sim \text{Poiss}\left(\sum_{b=1}^{B} \lambda_t^{(b)}\right).$$

{#eq=freq_n_bayes} Finally, for our prior $\pi(\underline{\lambda}_t)$, we first assume each parameter is independent, i.e.,

$$\pi(\underline{\lambda}_t) = \prod_{b=1}^{B} \pi(\lambda_t^{(b)}).$$

A reasonable prior for each $\lambda_t^{(b)}$ representing the expected number of reads for barcode b should span several orders of magnitude. Furthermore, we assume that no barcode in the dataset ever goes extinct. Thus, no frequency can equal zero, facilitating the computation of the log frequency ratios needed to infer the relative fitness. The log-normal distribution satisfies these constraints; therefore, for the prior, we assume

$$\lambda_t^{(b)} \sim \log\mathcal{N}(\mu_{\lambda_t^{(b)}}, \sigma_{\lambda_t^{(b)}}), \tag{S33}$$

with $\mu_{\lambda_t^{(b)}}, \sigma_{\lambda_t^{(b)}}$ as the user-defined parameters that characterize the prior distribution.

B.1.1. Summary

Putting all the pieces developed in this section together gives a term for our inference of the form

$$\pi(\underline{\underline{F}} \mid \underline{\underline{R}}) \propto \prod_{t=1}^{T} \left\{ \pi(\underline{r}_t \mid n_t, \underline{\lambda}_t) \pi(n_t \mid \underline{\lambda}_t) \left[\prod_{b=1}^{B} \pi(\lambda_t^{(b)}) \right] \right\} \tag{S34}$$

where

$$\underline{r}_t \mid n_t, \underline{\lambda}_t \sim \text{Multinomial}(n_t, \underline{f}_t(\underline{\lambda}_t)), \tag{S35}$$

$$n_t \mid \underline{\lambda}_t \sim \text{Poiss}\left(\sum_{b=1}^{B} \lambda_t^{(b)} \right). \tag{S36}$$

and

$$\lambda_t^{(b)} \sim \log \mathcal{N}(\mu_{\lambda_t^{(b)}}, \sigma_{\lambda_t^{(b)}}), \tag{S37}$$

B.2. Population mean fitness uncertainty $\pi(\underline{\bar{s}}_T \mid \underline{\underline{F}}, \underline{\underline{R}})$

Next, we turn our attention to the problem of determining the population mean fitnesses $\underline{\bar{s}}_T$. First, we notice that our fitness model in Equation 3 does not include the value of the raw reads. They enter the calculation indirectly through the inference of the frequency values we developed in Section B.1. This means that we can remove the conditioning of the value of $\underline{\bar{s}}_T$ on the number of reads, obtaining a simpler probability function

$$\pi(\underline{\bar{s}}_T \mid \underline{\underline{F}}, \underline{\underline{R}}) = \pi(\underline{\bar{s}}_T \mid \underline{\underline{F}}). \tag{S38}$$

Moreover, our fitness model does not directly explain how the population mean fitness evolves over time. In other words, our model cannot explicitly compute the population mean fitness at time $t+1$ from the information we have about time t. Given this model limitation, we are led to assume that we must infer each $\bar{s}_t$ independently. Expressing this for our inference results in

$$\pi(\underline{\bar{s}}_T \mid \underline{\underline{F}}) = \prod_{t=1}^{T-1} \pi(\bar{s}_t \mid \underline{f}_t, \underline{f}_{t+1}), \tag{S39}$$

where we split our matrix $\underline{\underline{F}}$ for each time point and only kept the conditioning on the relevant frequencies needed to compute the mean fitness at time t.

Although our fitness model in Equation 3 also includes the relative fitness $s^{(m)}$, to infer the population mean fitness we only utilize data from the neutral lineages that, by definition, have a relative fitness $s^{(n)} = 0$. Therefore, the conditioning on Equation S39 can be further simplified by only keeping the frequencies of the neutral lineages, i.e.,

$$\pi(\bar{s}_t \mid \underline{f}_t, \underline{f}_{t+1}) = \pi(\bar{s}_t \mid \underline{f}_t^N, \underline{f}_{t+1}^N). \tag{S40}$$

Recall that in Section 2.3 we emphasized that the frequencies $f_t^{(n)}$ do not represent the true frequency of a particular lineage in the population but rather a "normalized number of

cells." Therefore, it is safe to assume each of the N neutral lineages' frequencies is changing independently. The correlation of how increasing the frequency of one lineage will decrease the frequency of others is already captured in the model presented in Section B.1. Thus, we write

$$\pi(\bar{s}_t \mid \underline{f}_t^N, \underline{f}_{t+1}^N) = \prod_{n=1}^{N} \pi(\bar{s}_t \mid f_t^{(n)}, f_{t+1}^{(n)}). \tag{S41}$$

Now, we can focus on one of the terms on the right-hand side of Equation S41. Writing Bayes theorem results in

$$\pi(\bar{s}_t \mid f_t^{(n)}, f_{t+1}^{(n)}) \propto \pi(f_t^{(n)}, f_{t+1}^{(n)} \mid \bar{s}_t)\pi(\bar{s}_t). \tag{S42}$$

Notice the likelihood defines the joint distribution of neutral barcode frequencies conditioned on the population mean fitness. However, rewriting our fitness model in Equation 3 for a neutral lineage to leave frequencies on one side and fitness on the other results in

$$\frac{f_{t+1}^{(n)}}{f_t^{(n)}} = \mathrm{e}^{-\bar{s}_t \tau}. \tag{S43}$$

Equation S43 implies that our fitness model only relates **the ratio** of frequencies and not the individual values. To get around this complication, we define

$$\gamma_t^{(b)} \equiv \frac{f_{t+1}^{(b)}}{f_t^{(b)}}, \tag{S44}$$

as the ratio of frequencies between two adjacent time points for any barcode b. This allows us to rewrite the joint distribution $\pi(f_t^{(n)}, f_{t+1}^{(n)} \mid \bar{s}_t)$ as

$$\pi(f_t^{(n)}, f_{t+1}^{(n)} \mid \bar{s}_t) = \pi(f_t^{(n)}, \gamma_t^{(n)} \mid \bar{s}_t). \tag{S45}$$

Let us rephrase this subtle but necessary change of variables since it is a key part of the inference problem: our series of independence assumptions lead us to Equation S42 that relates the value of the population mean fitness $\bar{s}_t$ to the frequency of a neutral barcode at times t and $t+1$. However, as shown in Equation S43, our model functionally relates the ratio of frequencies—that we defined as $\gamma_t^{(n)}$—and not the independent frequencies to the mean fitness. Therefore, instead of writing for the likelihood the joint distribution of the frequency values at times t and $t+1$ conditioned on the mean fitness, we write the joint distribution of the barcode frequency at time t and the ratio of the frequencies. These **must be** equivalent joint distributions since there is a one-to-one mapping between $\gamma_t^{(n)}$ and $f_{t+1}^{(n)}$ for a given value of $f_t^{(n)}$. Another way to phrase this is to say that knowing the frequency at time t and at time $t+1$ provides the same amount of information as knowing the frequency at time t and the ratio of the frequencies. This is because if we want to obtain $f_{t+1}^{(n)}$ given this information, we simply compute

$$f_{t+1}^{(n)} = \gamma_t^{(n)} f_t^{(n)}. \tag{S46}$$

The real advantage of rewriting the joint distribution as in Equation S45 comes from splitting this joint distribution as a product of conditional distributions of the form

$$\pi(f_t^{(n)}, \gamma_t^{(n)} \mid \bar{s}_t) = \pi(f_t^{(n)} \mid \gamma_t^{(n)}, \bar{s}_t)\pi(\gamma_t^{(n)} \mid \bar{s}_t). \tag{S47}$$

Written in this form, we can finally propose a probabilistic model for how the mean fitness relates to the frequency ratios we determine in our experiments. The second term on the right-hand side of Equation S47 relates how the determined frequency ratio $\gamma_t^{(b)}$ relates to the mean fitness $\bar{s}_t$. From Equation S43 and Equation S44, we can write

$$\ln \gamma_t^{(n)} = -\bar{s}_t + \varepsilon_t^{(n)}, \tag{S48}$$

where, for simplicity, we set $\tau = 1$. Note that we added an extra term, $\varepsilon_t^{(n)}$, characterizing the deviations of the measurements from the theoretical model. We assume these errors are normally distributed with mean zero and some standard deviation σ_t, implying that

$$\ln \gamma_t^{(n)} \mid \bar{s}_t, \sigma_t \sim \mathcal{N}\left(-\bar{s}_t, \sigma_t\right), \tag{S49}$$

where we include the nuisance parameter σ_t to be determined. If we assume the log frequency ratio is normally distributed, this implies the frequency ratio itself is distributed log-normal. This means that

$$\gamma_t^{(n)} \mid \bar{s}_t, \sigma_t \sim \log\mathcal{N}\left(-\bar{s}_t, \sigma_t\right). \tag{S50}$$

Having added the nuisance parameter σ_t implies that we must update Equation S42 to

$$\pi(\bar{s}_t, \sigma_t \mid f_t^{(n)}, f_{t+1}^{(n)}) \propto \pi(f_t^{(n)}, \gamma_t^{(n)} \mid \bar{s}_t, \sigma_t)\pi(\bar{s}_t)\pi(\sigma_t), \tag{S51}$$

where we assume the prior for each parameter is independent, i.e.,

$$\pi(\bar{s}_t, \sigma_t) = \pi(\bar{s}_t)\pi(\sigma_t). \tag{S52}$$

For numerical stability, we will select weakly-informative priors for both of these parameters. In the case of the nuisance parameter σ_t, the prior must be restricted to positive values only, since standard deviations cannot be negative.

For the first term on the right-hand side of Equation S47, $\pi(f_t^{(n)} \mid \gamma_t^{(n)}, \bar{s}_t)$, we remove the conditioning on the population mean fitness since it does not add any information on top of what the frequency ratio $\gamma_t^{(n)}$ already gives. Therefore, we have

$$\pi(f_t^{(n)} \mid \gamma_t^{(n)}, \bar{s}_t) = \pi(f_t^{(n)} \mid \gamma_t^{(n)}). \tag{S53}$$

The right-hand side of Equation S53 asks us to compute the probability of observing a frequency value $f_t^{(n)}$ given that we get to observe the ratio $\gamma_t^{(n)}$. If the ratio happened to be $\gamma_t^{(n)} = 2$, we could have $f_{t+1}^{(n)} = 1$ and $f_{t+1}^{(n)} = 0.5$, for example. Although, it would be equally likely that $f_{t+1}^{(n)} = 0.6$ and $f_{t+1}^{(n)} = 0.3$ or $f_{t+1}^{(n)} = 0.1$ and $f_{t+1}^{(n)} = 0.05$ for that matter. If we only get to observe the frequency ratio $\gamma_t^{(n)}$, we know that the numerator $f_{t+1}^{(n)}$

can only take values between zero and one, all of them being equally likely given only the information on the ratio. As a consequence, the value of the frequency in the denominator $f_t^{(n)}$ is restricted to fall in the range

$$f_t^{(n)} \in \left(0, \frac{1}{\gamma_t^{(n)}}\right]. \tag{S54}$$

A priori, we do not have any reason to favor any value over any other, therefore it is natural to write

$$f_t^{(n)} \mid \gamma_t^{(n)} \sim \text{Uniform}\left(0, \frac{1}{\gamma_t^{(n)}}\right). \tag{S55}$$

B.2.1. Summary

Putting all the pieces we have developed in this section together results in an inference for the population mean fitness values of the form

$$\pi(\underline{\bar{s}}_T, \underline{\sigma}_T \mid \underline{\underline{F}}) \propto \prod_{t=1}^{T-1} \left\{ \prod_{n=1}^{N} \left[\pi(f_t^{(n)} \mid \gamma_t^{(n)}) \pi(\gamma_t^{(n)} \mid \bar{s}_t, \sigma_t)\right] \pi(\bar{s}_t)\pi(\sigma_t) \right\}, \tag{S56}$$

where we have

$$f_t^{(n)} \mid \gamma_t^{(n)} \sim \text{Uniform}\left(0, \frac{1}{\gamma_t^{(n)}}\right), \tag{S57}$$

$$\gamma_t^{(n)} \mid \bar{s}_t, \sigma_t \sim \log\mathcal{N}(\bar{s}_t, \sigma_t), \tag{S58}$$

$$\bar{s}_t \sim \mathcal{N}(0, \sigma_{\bar{s}_t}), \tag{S59}$$

and

$$\sigma_t \sim \log\mathcal{N}(\mu_{\sigma_t}, \sigma_{\sigma_t}), \tag{S60}$$

where $\sigma_{\bar{s}_t}$, μ_{σ_t}, and σ_{σ_t} are user-defined parameters.

B.3. Mutant relative fitness uncertainty $\pi(\underline{s}^M \mid \underline{\bar{s}}_T, \underline{\underline{F}}, \underline{\underline{R}})$

The last piece of our inference is the piece that we care about the most: the probability distribution of all the mutants' relative fitness, given the inferred population mean fitness and the frequencies. First, we assume that all fitness values are independent of each other. This allows us to write

$$\pi(\underline{s}^M \mid \underline{\bar{s}}_T, \underline{\underline{F}}, \underline{\underline{R}}) = \prod_{m=1}^{M} \pi(s^{(m)} \mid \underline{\bar{s}}_T, \underline{\underline{F}}, \underline{\underline{R}}). \tag{S61}$$

Furthermore, as was the case with the population mean fitness, our fitness model relates frequencies, not raw reads. Moreover, the fitness value of mutant m only depends on the frequencies of such mutant. Therefore, we can simplify the conditioning to

$$\pi(s^{(m)} \mid \underline{\bar{s}}_T, \underline{\underline{F}}, \underline{\underline{R}}) = \pi(s^{(m)} \mid \underline{\bar{s}}_T, \underline{f}^{(m)}), \tag{S62}$$

where

$$\underline{f}^{(m)} = (f_0^{(m)}, f_1^{(m)}, \dots, f_T^{(m)})^\dagger, \tag{S63}$$

is the vector containing the frequency time series for mutant m. Writing Bayes' theorem for the right-hand side of Equation S62 results in

$$\pi(s^{(m)} \mid \underline{\bar{s}}_T, \underline{f}^{(m)}) \propto \pi(\underline{f}^{(m)} \mid \underline{\bar{s}}_T, s^{(m)})\pi(s^{(m)} \mid \underline{\bar{s}}_T). \tag{S64}$$

Notice the conditioning on the mean fitness values $\underline{\bar{s}}_T$ is not inverted since we already inferred these values.

Following the logic used in Section B.2, let us define

$$\underline{\gamma}^{(m)} = (\gamma_0^{(m)}, \gamma_1^{(m)}, \dots, \gamma_{T-1}^{m})^\dagger, \tag{S65}$$

where each entry $\gamma_t^{(m)}$ is defined by Equation S44. In the same way we rewrote the joint distribution between two adjacent time point frequencies to the joint distribution between one of the frequencies and the ratio of both frequencies in Equation S45, we can rewrite the joint distribution of the frequency time series for mutant m as

$$\pi(\underline{f}^{(m)} \mid \underline{\bar{s}}_T, s^{(m)}) = \pi(f_0^{(m)}, \underline{\gamma}^{(m)} \mid \underline{\bar{s}}_T, s^{(m)}). \tag{S66}$$

One can think about Equation S66 as saying that knowing the individual frequencies at each time point contain equivalent information as knowing the initial frequency and the subsequent ratios of frequencies. This is because if we want to know the value of $f_1^{(m)}$ given the ratios, we only need to compute

$$f_1^{(m)} = \gamma_0^{(m)} f_0^{(m)}. \tag{S67}$$

Moreover, if we want to know $f_2^{(m)}$, we have

$$f_2^{(m)} = \gamma_1^{(m)} f_1^{(m)} = \gamma_1^{(m)} \left(\gamma_0^{(m)} f_0^{(m)}\right), \tag{S68}$$

and so on. We can then write the joint distribution on the right-hand side of Equation S66 as a product of conditional distributions of the form

$$\begin{aligned}\pi(f_0^{(m)}, \underline{\gamma}^{(m)} \mid \underline{\bar{s}}_T, s^{(m)}) =& \pi(f_0^{(m)} \mid \gamma_0^{(m)}, \dots, \gamma_{T-1}^{(m)}, \underline{\bar{s}}_T, s^{(m)}) \times \\ & \pi(\gamma_0^{(m)} \mid \gamma_1^{(m)}, \dots, \gamma_{T-1}^{(m)}, \underline{\bar{s}}_T, s^{(m)}) \times \\ & \pi(\gamma_1^{(m)} \mid \gamma_2^{(m)}, \dots, \gamma_{T-1}^{(m)}, \underline{\bar{s}}_T, s^{(m)}) \times \\ & \vdots \\ & \pi(\gamma_{T-2}^{(m)} \mid \gamma_{T-1}^{(m)}, \underline{\bar{s}}_T, s^{(m)}) \times \\ & \pi(\gamma_{T-1}^{(m)} \mid \underline{\bar{s}}_T, s^{(m)}).\end{aligned} \tag{S69}$$

Writing the fitness model in Equation 3 as

$$\gamma_t^{(m)} = \frac{f_{t+1}^{(m)}}{f_t^{(m)}} = \mathrm{e}^{(s^{(m)} - s_t)\tau},$$

reveals that the value of each of the ratios $\gamma_t^{(m)}$ only depends on the corresponding fitness value $\bar{s}_t$ and the relative fitness $s^{(m)}$. Therefore, we can remove most of the conditioning on the right-hand side of Equation S69, resulting in a much simpler joint distribution of the form

$$\begin{aligned}\pi(f_0^{(m)}, \underline{\gamma}^{(m)} \mid \underline{\bar{s}}_T, s^{(m)}) = &\pi(f_0^{(m)} \mid \gamma_0^{(m)}) \times \\ &\pi(\gamma_0^{(m)} \mid \bar{s}_0, s^{(m)}) \times \\ &\pi(\gamma_1^{(m)} \mid \bar{s}_1, s^{(m)}) \times \\ &\vdots \\ &\pi(\gamma_{T-2}^{(m)} \mid \bar{s}_{T-2}, s^{(m)}) \times \\ &\pi(\gamma_{T-1}^{(m)} \mid \bar{s}_{T-1}, s^{(m)}),\end{aligned} \tag{S70}$$

where for the first term on the right-hand side of Equation S70 we apply the same logic as in Equation S53 to remove all other dependencies. We emphasize that although Equation S70 looks like a series of independent inferences, the value of the relative fitness $s^{(m)}$ is shared among all of them. This means that the parameter is not inferred individually for each time point, resulting in different estimates of the parameter, but each time point contributes independently to the inference of a single estimate of $s^{(m)}$.

Using equivalent arguments to those in Section B.2, we assume

$$f_0^{(m)} \mid \gamma_0^{(m)} \sim \text{Uniform}\left(0, \frac{1}{\gamma_0^{(m)}}\right),$$

and

$$\gamma_t^{(m)} \mid \bar{s}_t, s^{(m)}, \sigma^{(m)} \sim \log\mathcal{N}\left(s^{(m)} - \bar{s}_t, \sigma^{(m)}\right), \tag{S71}$$

where we add the nuisance parameter $\sigma^{(m)}$ to the inference. Notice that this parameter is not indexed by time. This means that we assume the deviations from the theoretical prediction do not depend on time, but only on the mutant. Adding the nuisance parameter demands us to update Equation S64 to

$$\pi(s^{(m)}, \sigma^{(m)} \mid \underline{\bar{s}}_T, \underline{f}^{(m)}) \propto \pi(\underline{f}^{(m)} \mid \underline{\bar{s}}_T, s^{(m)}, \sigma^{(m)})\pi(s^{(m)})\pi(\sigma^{(m)}), \tag{S72}$$

where we assume independent priors for both parameters. We also removed the conditioning on the values of the mean fitness as knowing such values does not change our prior information about the possible range of values that the parameters can take. As with the priors on Section B.2, we will assign weakly-informative priors to these parameters.

B.3.1. Summary

With all pieces in place, we write the full inference of the relative fitness values as

$$\pi(\underline{s}^M, \underline{\sigma}^M \mid \underline{\bar{s}}_T, \underline{\underline{F}}) \propto \prod_{m=1}^{M} \left\{ \pi(f_0^{(m)} \mid \gamma_0^{(m)}) \prod_{t=0}^{T-1} \left[\pi(\gamma_t^{(m)} \mid \bar{s}_t, s^{(m)}, \sigma^{(m)}) \right] \pi(s^{(m)}) \pi(\sigma^{(m)}) \right\}, \tag{S73}$$

where

$$f_0^{(m)} \mid \gamma_0^{(m)} \sim \text{Uniform}\left(0, \frac{1}{\gamma_0^{(m)}}\right), \tag{S74}$$

$$\gamma_t^{(m)} \mid \bar{s}_t, s^{(m)}, \sigma^{(m)} \sim \log \mathcal{N}\left(s^{(m)} - \bar{s}_t, \sigma^{(m)}\right), \tag{S75}$$

$$s^{(m)} \sim \mathcal{N}(0, \sigma_{s^{(m)}}), \tag{S76}$$

and

$$\sigma^{(m)} \sim \log \mathcal{N}(\mu_{\sigma^{(m)}}, \sigma_{\sigma^{(m)}}), \tag{S77}$$

where $\sigma_{s^{(m)}}$, $\mu_{\sigma^{(m)}}$, and $\sigma_{\sigma^{(m)}}$ are user-defined parameters.

B.4. Hierarchical models for multiple experimental replicates

As detailed in Section 2.7 of the main text, we define a Bayesian hierarchical model to analyze data from multiple experimental replicates. The implementation requires only slightly modifying the base model detailed in the previous sections. The hierarchical model defines a hyper-fitness parameter $\theta^{(m)}$ for every non-neutral barcode. We can thus collect all of the M hyperparameters in an array of the form

$$\underline{\theta}^M = (\theta^{(1)}, \dots, \theta^{(M)})^\dagger. \tag{S78}$$

Our data now consists of a series of matrices $\underline{\underline{R}}_{[j]}$, where the subindex $[j]$ refers to the j-th experimental replicate. These matrices need not have the same number of rows, as the time points measured for each replicate can vary. The statistical model we must define is then of the form

$$\begin{aligned} \pi(\underline{\theta}^M, \{\underline{s}^M_{[j]}\}, \{\underline{\bar{s}}_{T[j]}\}, \{\underline{\underline{F}}_{[j]}\} \mid \{\underline{\underline{R}}_{[j]}\}) \propto \pi(\{\underline{\underline{R}}_{[j]}\} \mid \underline{\theta}^M, \{\underline{s}^M_{[j]}\}, \{\underline{\bar{s}}_{T[j]}\}, \{\underline{\underline{F}}_{[j]}\}) \times \\ \pi(\underline{\theta}^M, \{\underline{s}^M_{[j]}\}, \{\underline{\bar{s}}_{T[j]}\}, \{\underline{\underline{F}}_{[j]}\}) \end{aligned} \tag{S79}$$

where the parameters within curly braces with subindex $[j]$ indicate one set of parameters per experimental replicate. For example,

$$\{\underline{s}^M_{[j]}\} = \{\underline{s}^M_{[1]}, \underline{s}^M_{[2]}, \dots \underline{s}^M_{[E]}\}, \tag{S80}$$

where E is the number of experimental replicates.

Given the dependencies between the variables, we can factorize Equation S79 to be of the form

$$
\begin{aligned}
\pi(\underline{\theta}^M, \{\underline{s}^M_{[j]}\}, \{\underline{\bar{s}}_{T[j]}\}, \{\underline{\underline{F}}_{[j]}\} \mid \{\underline{\underline{R}}_{[j]}\}) =& \pi(\underline{\theta}^M, \{\underline{s}^M_{[j]}\} \mid \{\underline{\bar{s}}_{T[j]}\}, \{\underline{\underline{F}}_{[j]}\}) \times \\
& \pi(\{\underline{\bar{s}}_{T[j]}\} \mid \{\underline{\underline{F}}_{[j]}\}) \times \\
& \pi(\{\underline{\underline{F}}_{[j]}\} \mid \{\underline{\underline{R}}_{[j]}\})
\end{aligned}
\tag{S81}
$$

Furthermore, the hierarchical structure only connects the replicates via the relative fitness parameters. This means that the population mean fitness values and the frequencies can be independently inferred for each dataset. This allows us to rewrite the right-hand side of Equation S81 as

$$
\begin{aligned}
\pi(\underline{\theta}^M, \{\underline{s}^M_{[j]}\}, \{\underline{\bar{s}}_{T[j]}\}, \{\underline{\underline{F}}_{[j]}\} \mid \{\underline{\underline{R}}_{[j]}\}) =& \pi(\underline{\theta}^M, \{\underline{s}^M_{[j]}\} \mid \{\underline{\bar{s}}_{T[j]}\}, \{\underline{\underline{F}}_{[j]}\}) \times \\
& \prod_{j=1}^{E} \left[\pi(\underline{\bar{s}}_{T[j]} \mid \underline{\underline{F}}_{[j]}) \pi(\underline{\underline{F}}_{[j]} \mid \underline{\underline{R}}_{[j]}) \right].
\end{aligned}
\tag{S82}
$$

The terms inside the square brackets in Equation S82 take the same functional form as those derived in Section B.2 and Section B.1. Therefore, to implement the desired hierarchical model, we only need to focus on the first term on the right-hand side of Equation S82. A way to think about the structure of the hierarchical model is as follows: imagine each genotype as a "*true*" relative fitness value. However, every time we perform an experiment, small variations in the biotic and abiotic conditions—also known as batch effects—might result in small deviations from this value. We model this by defining a distribution for the hyper-fitness parameter—the ground truth we are interested in—and having each experimental replicate sample from this hyper-parameter distribution to determine the "*local*" fitness value. The wider the hyper-parameter distribution is the more variability between experimental replicates.

Writing Bayes' theorem for the first term in Equation S82 results in

$$
\pi(\underline{\theta}^M, \{\underline{s}^M_{[j]}\} \mid \{\underline{\underline{F}}_{[j]}\}, \{\underline{\bar{s}}_{T[j]}\}) \propto \pi(\{\underline{\underline{F}}_{[j]}\} \mid \underline{\theta}^M, \{\underline{s}^M_{[j]}\}, \{\underline{\bar{s}}_{T[j]}\}) \pi(\underline{\theta}^M, \{\underline{s}^M_{[j]}\} \mid \{\underline{\bar{s}}_{T[j]}\}),
\tag{S83}
$$

where we leave the conditioning on the population mean fitness as we did in Section B.3. This expression can be simplified in two ways. First, the frequency values for each experimental replicate depend directly on the local fitness values and the corresponding population mean fitness, as the relationship between experimental replicates only occurs through the relative fitness values. Therefore, we can write

$$
\pi(\underline{\theta}^M, \{\underline{s}^M_{[j]}\} \mid \{\underline{\underline{F}}_{[j]}\}, \{\underline{\bar{s}}_{T[j]}\}) \propto \prod_{j=1}^{E} \left[\pi(\underline{\underline{F}}_{[j]} \mid \underline{s}^M_{[j]}, \underline{\bar{s}}_{T[j]}) \right] \pi(\underline{\theta}^M, \{\underline{s}^M_{[j]}\} \mid \{\underline{\bar{s}}_{T[j]}\}).
\tag{S84}
$$

Second, the relationship between the hyper-fitness and the local fitness values allows us to write their joint distribution as a conditional distribution where local fitness values depend

on the global hyper-fitness value, obtaining

$$\pi(\underline{\theta}^M, \{\underline{s}^M_{[j]}\} \mid \{\underline{\underline{F}}_{[j]}\}, \{\underline{\bar{s}}_{T[j]}\}) \propto \prod_{j=1}^{E} \left[\pi(\underline{\underline{F}}_{[j]} \mid \underline{s}^M_{[j]}, \underline{\bar{s}}_{T[j]})\pi(\underline{s}^M_{[j]} \mid \underline{\theta}^M)\right] \pi(\underline{\theta}^M). \tag{S85}$$

Notice we removed the conditioning on the population mean fitness as our prior expectations of what the global hyper-fitness or local fitness value might be do not depend on these nuisance parameters.

The first term on the right-hand side of Equation S85 takes the same functional form as the one derived in Section B.3. Therefore, all we are left with is to determine the functional forms for the hyper–prior $\pi(\underline{\theta}^M)$, and the conditional probability $\pi(\underline{s}^M_{[j]} \mid \underline{\theta}^M)$. In analogy to the assumptions used for the fitness values in Section B.3, we define the value of each hyper-fitness as independent. This means that we have

$$\pi(\underline{\theta}^M) = \prod_{m=1}^{M} \pi(\theta^{(m)}). \tag{S86}$$

Furthermore, we assume this prior is of the form

$$\theta^{(m)} \sim \mathcal{N}(\mu_{\theta^{(m)}}, \sigma_{\theta^{(m)}}), \tag{S87}$$

where $\mu_{\theta^{(m)}}$ and $\sigma_{\theta^{(m)}}$ are user-defined parameters encoding the prior expectations on the fitness values.

For the conditional distribution $\pi(\underline{s}^M_{[j]} \mid \underline{\theta}^M)$, we use the so-called non-centered parametrization that avoids some of the intrinsic degeneracies associated with hierarchical models [4]. We invite the reader to check this excellent blog explaining the difficulties of working with hierarchical models. This non-centered parameterization implies that we introduce two nuisance parameters such that the local fitness $s^{(m)}_{[j]}$ is computed as

$$s^{(m)}_{[j]} = \theta^{(m)} + (\tau^{(m)}_{[j]} \times \xi^{(m)}_{[j]}), \tag{S88}$$

where $\theta^{(m)}$ is the corresponding genotype hyper-fitness value, $\xi^{(m)}_{[j]}$ is a standard normal random variable, i.e.,

$$\xi^{(m)}_{[j]} \sim \mathcal{N}(0, 1), \tag{S89}$$

that allows deviations from the hyper-fitness value to be either positive or negative, and $\tau^{(m)}_{[j]}$ is a strictly positive random variable that characterizes the deviation of the local fitness value from the global hyper-fitness. We assume

$$\tau^{(m)}_{[j]} \sim \log\mathcal{N}(\mu_{\tau^{(m)}_{[j]}}, \sigma_{\tau^{(m)}_{[j]}}) \tag{S90}$$

where $\mu_{\tau^{(m)}_{[j]}}$ and $\sigma_{\tau^{(m)}_{[j]}}$ are user-defined parameters capturing the expected magnitude of the batch effects.

B.5. Defining prior probabilities

One aspect commonly associated—in both positive and negative ways—to Bayesian analysis is the definition of prior probabilities. On the one hand, the naive textbook version of Bayesian analysis defines the prior as encoding the information we have about the inference in question before acquiring any data. This is the "ideal" use of priors that, whenever possible, should be implemented. On the other hand, for most practitioners of Bayesian statistics in the age of big data, the definition of prior becomes a tool to ensure the convergence of sampling algorithms such as MCMC [5]. However, for our particular problem, although we deal with large amounts of data (inferences can be made for $>$ 10K barcodes over multiple time points, resulting in $>$ 100K parameters), each barcode has very little data, as they are measured only once per time point over $<$ 10 growth-dilution cycles. Furthermore, it is incredibly challenging to understand the noise sources related to culturing conditions, DNA extraction, library preparation, etc., and encode them into reasonable prior distributions.

Empirically, our approach for this work defined the priors based solely on the neutral lineage data, as they represent the only repeated measurements of a single genotype in our experimental design. We acknowledge that defining the priors after observing the data might be considered an incoherent inference. However, as expressed by Gelman, Simpson, and Betancourt [5]

> Incoherence is an unavoidable aspect of much real-world data analysis; and, indeed, one might argue that as scientists we learn the most from the anomalies and reassessments associated with episodes of incoherence.

With this in mind, we leave it to the reader to judge the selection of priors. Furthermore, the software package associated with this work, `BarBay.jl`, is written so that users can experiment with different prior selection criteria that fit their needs. We strongly advocate that statistics should not be done in a black-box fit-all tool mindset but rather as a formal way to encode the assumptions behind the analysis, subject to constructive criticism. With this philosophical baggage behind us, let us now focus on how the priors used for this work were selected.

B.5.1. Naive neutral lineage-based priors

For the base model presented in this work, the user-defined prior parameters include the following:

- Prior on population mean fitness (one per pair of adjacent time points)

$$\bar{s}_t \sim \mathcal{N}(\mu_{\bar{s}_t}, \sigma_{\bar{s}_t}). \tag{S91}$$

- Prior on standard deviation associated with neutral lineages likelihood function (one per pair of adjacent time points)

$$\sigma_t \sim \log\mathcal{N}(\mu_{\sigma_t}, \sigma_{\sigma_t}). \tag{S92}$$

- Prior on relative fitness (one per non-neutral barcode)

$$s^{(m)} \sim \mathcal{N}(\mu_{s^{(m)}}, \sigma_{s^{(m)}}). \tag{S93}$$

- Prior on standard deviation associated with non-neutral lineages likelihood function (one per non-neutral barcode)

$$\sigma^{(m)} \sim \log\mathcal{N}(\mu_{\sigma^{(m)}}, \sigma_{\sigma^{(m)}}) \tag{S94}$$

The `BarBay.jl` package includes a function `naive_prior` within the `stats` module. This function utilizes the data from the neutral lineages to determine some of the prior parameters to facilitate the inference algorithm's numerical convergence. In particular, it defines the population mean fitness parameter $\mu_{\bar{s}_t}$ as

$$\mu_{\bar{s}_t} = \frac{1}{N}\sum_{n=1}^{N} -\ln\left(\frac{r_{t+1}^{(n)}}{r_t^{(n)}}\right), \tag{S95}$$

where N is the number of neutral lineages and $r_t^{(n)}$ is the number of neutral lineages. In other words, it defines the mean of the prior distribution as the mean of what one naively would compute from the neutral lineages, discarding cases where the ratio diverges because the denominator $r_t^{(n)} = 0$. For the variance parameter, we chose a value $\sigma_{\bar{s}_t} = 0.05$.

Furthermore, the `naive_prior` function defines the mean of the variance parameter as the standard deviation of the log frequency ratios for the neutral lineages, i.e.,

$$\mu_{\sigma_t} = \sqrt{\mathrm{Var}\left(\frac{r_{t+1}^{(n)}}{r_t^{(n)}}\right)}, \tag{S96}$$

where Var is the sample variance. This same value was utilized for the mean of the non-neutral barcode variance $\mu_{\sigma^{(m)}}$. While we assigne the corresponding variances to be $\sigma_{\sigma_t} = \sigma_{\sigma^{(m)}} = 1$.

C. Posterior predictive checks

Throughout the main text, we allude to the concept of posterior predictive checks as a formal way to assess the accuracy of our inference pipeline. Here, we explain the mechanics behind the computation of these credible regions, given the output of the inference.

Bayesian models encode what is known as a *generative model*. This statement means that in our definition of the likelihood function and the prior distribution, we, as modelers, propose a mathematical function that captures all relevant relationships between unobserved (latent) variables. Therefore, when these latent variables are input into the mathematical model, this function *generates* data that should be, in principle, indistinguishable from the real

observations if the model is a good account of the underlying processes involved in the phenomena of interest. This generative model implies that once we run the inference process and update our posterior beliefs about the state of the latent variables, we can input back the inferred values to our model and generate synthetic data. Furthermore, we can repeat this process multiple times to compute the range where we expect to observe our data conditioned on the accuracy of the model.

For our specific scenario, recall that our objective is to infer the relative fitness of a non-neutral lineage $s^{(m)}$ along with nuisance parameters related to the population mean fitness at each point, $\underline{\bar{s}}_t$, and the barcode frequency time series $\underline{f}^{(m)}$. All these variables are related through our fitness model (see Section 2.3 in the main text)

$$f_{t+1}^{(m)} = f_t^{(m)} \mathrm{e}^{(s^{(m)} - s_t)\tau}. \tag{S97}$$

As we saw, it is convenient to rewrite Equation S97 as

$$\frac{1}{\tau} \ln \frac{f_{t+1}^{(m)}}{f_t^{(m)}} = (s^{(m)} - s_t). \tag{S98}$$

Written in this way, we separate the quantities we can compute from the experimental observations—the left-hand side of Equation S98 can be computed from the barcode reads—from the latent variables.

Although we perform the joint inference over all barcodes in the present work, let us focus on the inference task for a single barcode as if it were computed independently. For a non-neutral barcode, our task consists of computing the posterior probability

$$\pi(\theta \mid \underline{r}^{(m)}) = \pi(s^{(m)}, \sigma^{(m)}, \underline{s}_t, \underline{f}^{(m)} \mid \underline{r}^{(m)}), \tag{S99}$$

where θ represents all parameters to be inferred and $\underline{r}^{(m)}$ is the vector with the barcode raw counts time series. The list of parameters are

- $s^{(m)}$: The barcode's relative fitness.
- $\sigma^{(m)}$: A nuisance parameter used in the likelihood to generate the data. This captures the expected deviation from Equation S98
- $\underline{s}_t$: The vector with all population mean fitness for each pair of adjacent time points.
- $\underline{f}^{(m)}$: The vector with the barcode frequency time series.

Furthermore, let us define a naive estimate of the barcode frequency at time t as

$$\hat{f}_t^{(m)} = \frac{r_t^{(m)}}{\sum_{b=1}^{B} r_t^{(b)}}. \tag{S100}$$

We can compute this quantity from the data by normalizing the raw barcode counts by the sum of all barcode counts. Furthermore, we can compute a naive estimate of the log

frequency ratio from the raw barcode counts as

$$\ln \hat{\gamma}_t^{(m)} = \ln \frac{\hat{f}_{t+1}^{(m)}}{\hat{f}_t^{(m)}} \tag{S101}$$

In our generative model, we assumed

$$\ln \gamma_t^{(m)} \mid \theta \sim \mathcal{N}(s^{(m)} - s_t, \sigma^{(m)}). \tag{S102}$$

This implies that once we determine the posterior distribution of our parameters, we can generate synthetic values of $\ln \gamma_t^{(m)}$ that we can then compare with the values obtained from applying Equation S101 and Equation S101 to the raw data.

In practice, to compute the posterior predictive checks, we generate multiple samples from the posterior distribution $\pi(\theta \mid \underline{r}^{(m)})$

$$\underline{\theta} = (\theta_1, \theta_2, \ldots, \theta_N). \tag{S103}$$

With these samples in hand, the `BarBay.jl` package includes the function `logfreq_ratio_bc_ppc` for non-neutral barcodes that uses this set of posterior parameter samples to generate samples from the distribution defined in Equation S102. For a large-enough number of samples, we can then compute the desired percentiles—5, 68, and 95 percentiles in all figures in the main text—that are equivalent to the corresponding credible regions. In other words, the range of values of $\ln \gamma_t^{(m)}$ generated by this bootstrap process can be used to compute the region where we expect to find our raw estimates $\ln \hat{\gamma}_t^{(m)}$ with the desired probability. The package `BarBay.jl` includes an equivalent function, `logfreq_ratio_popmean_ppc`, for neutral lineages.

D. Logistic growth simulation

In this section, we explain the simulations used to assess the validity of our inference pipeline. Let us begin by assuming that, since the strains are grown for two full days in the experiment, having left behind the exponential phase for almost an entire day, a simple exponential growth of the form

$$\frac{dn_i}{dt} = \lambda_i n_i, \tag{S104}$$

where n_i is the number of cells of strain i, and λ_i is the corresponding growth rate is not enough. Instead, we will assume that the cells follow the logistic growth equation of the form

$$\frac{dn_i}{dt} = \lambda_i n_i \left(1 - \frac{\sum_{j=1}^{N} n_i}{\kappa}\right), \tag{S105}$$

where κ is the carrying capacity, and N is the total number of strains in the culture.

The inference method is based on the model that assumes that the time passed between dilutions $\tau \approx 8$ generations, the change in frequency for a mutant barcode can be approximated from cycle t to the next cycle $t+1$ as

$$f_{t+1}^{(m)} = f_t^{(m)} \mathrm{e}^{(s^{(m)} - \bar{s}_t)\tau}, \tag{S106}$$

where $s^{(m)}$ is the relative fitness for strain i compared to the ancestral strain and $\bar{s}_t$ is the mean fitness of the population at cycle t. To test this assumption, we implemented a numerical experiment following the logistic growth model described in Equation S105. Figure B shows an example of the deterministic trajectories for 50 labeled neutral lineages and 1000 lineages of interest. The upper red curve that dominates the culture represents the unlabeled ancestral strain included in the experimental design described in Section 2.1.

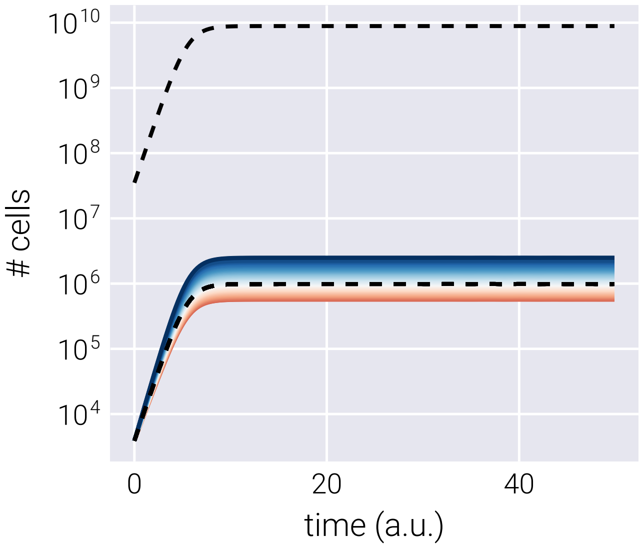

Figure B. Logistic growth simulation over single growth cycle. The dashed line represents the neutral lineages, with the upper curve being the unlabeled neutral strain. Color curves represent the genotypes of interest colored by growth rate relative to the neutral lineage.

To simulate multiple growth-dilution cycles, we take the population composition at the final time point and use it to initialize a new logistic growth simulation. Figure C shows the resulting number of cells at the last time point of a cycle over multiple growth-dilution cycles for the genotypes in @Figure B. We can see that the adaptive lineages (blue curves) increase in abundance, while detrimental lineages (red curves) decrease.

In Section 2.3, we derive the functional form to infer the relative fitness of each lineage as

$$\frac{1}{\tau} \ln \frac{f_{t+1}^{(b)}}{f_t^{(b)}} = (s^{(b)} - \bar{s}_t). \tag{S107}$$

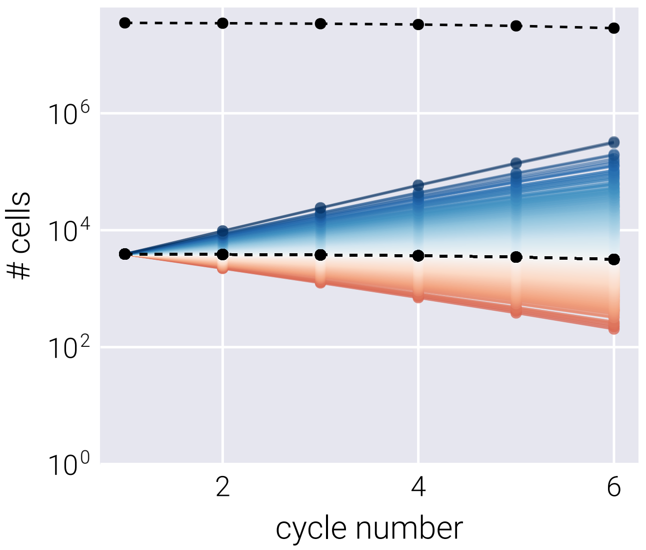

Figure C. Growth-dilution cycles for logistic growth simulation. Each point represents the final number of cells after a growth cycle for each lineage. Colors are the same as in Figure B.

Figure D shows the corresponding log frequency ratio curves for the logistic growth simulation. The displacement of these curves with respect to the neutral lineages determines the ground truth relative fitness value for these simulations.

To simulate the experimental noise, we add two types of noise:

1. Poisson noise between dilutions. For this, we take the final point of the logistic growth simulation and sample a random Poisson number based on this last point to set the initial condition for the next cycle.
2. Gaussian noise when performing the measurements. When translating the underlying population composition to the number of reads, we can add a custom amount of Gaussian noise.

Figure E shows the frequency trajectories (left panels) and log frequency ratios (right panels) for a noiseless simulation (upper panels) and a simulation with added noise (lower panels). The noiseless simulation is used to determine the relative fitness for each of the lineages, which serves as the ground truth to be compared with the resulting inference.

D.1. Simulation details

This section lists the parameters used to generate the synthetic data used throughout the main text. All the code to reproduce every simulation and figure in the paper can be found

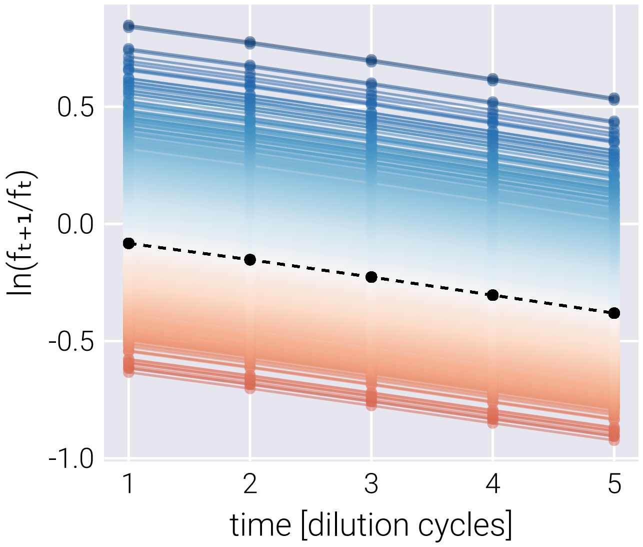

Figure D. Log frequency ratio for logistic growth simulations. The relative distance of the color curves from the black dashed line determines the relative fitness of each linage.

in the GitHub repository. Unless stated otherwise, the simulations were initialized following these steps:

1. The initial number of cells were defined as:

- Ancestor lineage: `n_init` $\times$ `frac_anc`.
- Neutral lineages: $n_0^{(n)} \sim$ Poisson $\left(\texttt{n\_init} \times \frac{\texttt{frac\_neutral}}{\texttt{n\_neutral}}\right)$
- Mutant lineages: $n_0^{(m)} \sim$ LogNormal $\left(\mu = \log(\texttt{n\_init} \times \frac{\texttt{frac\_mut}}{\texttt{n\_mut}}), \sigma = 2\right)$

where

- `n_init` is the initial number of cells.
- `frac_anc` $\in [0, 1]$ is the initial fraction of the culture that is ancestor.
- `frac_neutral` $\in [0, 1]$ is the initial fraction of the culture that is barcoded neutral lineages.
- `frac_mut` $\in [0, 1]$ is the initial fraction of the culture that is barcoded lineages whose relative fitness we want to infer–hereafter "mutat" barcodes.
- `frac_anc` $+$ `frac_neutral` $+$ `frac_mut` $= 1$
- `n_neutral` is the number of unique neutral lineage barcodes.
- `n_mut` is the number of unique mutant barcodes.

2. The distribution of growth rates λ were defined as

- Ancestor lineage & neutral lineages: $\lambda_a =$ _a. See details below for specific values for each simulation.

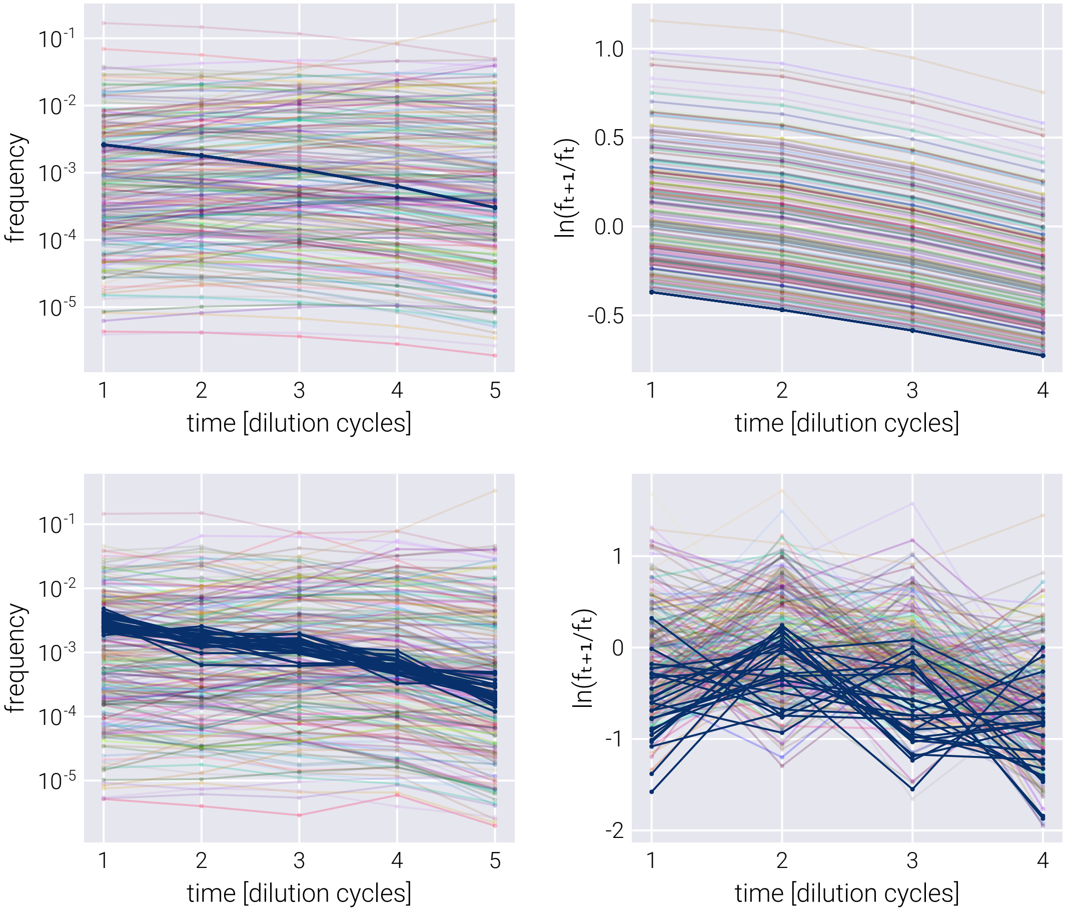

Figure E. Logistic growth-dilution simulations with and without noise

- Mutant lineages: $\lambda_m \sim$ truncated $- \mathcal{N}(\mu =$ `_mut_mean`, $\sigma =$ `_bc_std`, truncation limits $=$ `_trunc`). See details below for specific values for each simulation.

D.1.1. Fig 2.

The following is a `README.md` file automatically generated when simulating the data for Fig. 2 in the main text:

- File location `link to file`
- Number of mutant barcodes `n_mut = 225`
- Number of neutral barcodes `n_neutral = 25`
- Ancestral strain growth rate `_a = 1.0`
- Carrying capacity `= 1.0e10`
- Number of generations `n_gen = 8`
- Initial number of cells `n_init = / (2^(n_gen)) = 3.90625e7`
- Initial fraction of culture that is ancestor `frac_anc = 0.93`
- Initial fraction of culture that is neutrals `frac_neutral = 0.02`
- Initial fraction of culture that is mutants `frac_mut = 0.04999999999999995`
- Mutant fitness distribution mean `_mut_mean = _a * 1.005 = 1.005`
- Mutant fitness distribution standard deviation to sample growth rates `_mut_std = 0.1`
- Mutant fitness distribution truncation ranges for growth rates `_trunc = [_a .* 0.9999, _a * 1.5] = [0.9999, 1.5]`
- Gaussian noise distribution standard deviation `_lognormal = 0.3`

D.1.2. Fig 3.

The following is a `README.md` file automatically generated when simulating the data for Fig. 3 in the main text:

- File location `link to file`
- Number of environments `n_env = 3`
- Environment transitions `env_idx = [1, 2, 3, 1, 2, 3]`
- Number of mutant barcodes `n_mut = 900`
- Number of neutral barcodes `n_neutral = 100`
- Ancestral strain growth rate on each environment `_a = [1.0, 2.0, 0.5]`
- Carrying capacity `= 1.0e10`
- Number of generations `n_gen = 8`
- Initial number of cells `n_init = / (2^(n_gen)) = 3.90625e7`
- Initial fraction of culture that is ancestor `frac_anc = 0.93`
- Initial fraction of culture that is neutrals `frac_neutral = 0.02`
- Initial fraction of culture that is mutants `frac_mut = 0.04999999999999995`
- Mutant fitness distribution mean ‘ _mut_mean = _a * 1.005 = [1.005, 2.01, 0.5025]

- Mutant fitness distribution standard deviation to sample growth rates _mut_std = 0.1
- Mutant fitness distribution truncation ranges for growth rates _trunc = [_a .* 0.9995, _a .* 1.5] = [[[0.995, 1.99, 0.4975]], [[1.5, 3.0, 0.75]]]
- Gaussian noise distribution standard deviation _lognormal = 0.125

The variable env_idx lists the order of the environment cycles used in the simulation. In this case, transitions were of the form 1 -> 2, 2 -> 3, and 3 -> 1.

D.1.3. Fig 4.

The following is a README.md file automatically generated when simulating the data for Fig. 4 in the main text:

- File location link to file
- Number of experimental replicates n_data = 2
- Number of mutant barcodes n_mut = 900
- Number of neutral barcodes n_neutral = 100
- Ancestral strain growth rate _a = 1.0
- Carrying capacity = 1.0e10
- Number of generations n_gen = 8
- Initial number of cells n_init = / (2^(n_gen)) = 3.90625e7
- Initial fraction of culture that is ancestor frac_anc = 0.93
- Initial fraction of culture that is neutrals frac_neutral = 0.02
- Initial fraction of culture that is mutants frac_mut = 0.04999999999999995
- Mutant fitness distribution mean _bc_mean = _a * 1.005 = 1.005
- Mutant fitness distribution standard deviation to sample growth rates _bc_std = 0.1
- Mutant fitness distribution truncation ranges for growth rates _trunc = [_a .* 0.9999, _a * 1.5] = [0.9999, 1.5]
- Gaussian noise distribution standard deviation _lognormal = 0.3
- Standard deviation for between-experiment fitness distribution variability _exp = 0.015

The variable _exp defines the variance used to sample the variability between simulated experimental replicates. In other words, given a "hyper-fitness" value λ, for each experimental replicate, the growth rate was defined as $\lambda_j \sim \mathcal{N}(\lambda,$ _exp $)$.

D.1.4. Fig 6.

The following is a README.md file automatically generated when simulating the data for Fig. 6 in the main text:

- File location link to file
- Number of mutant barcodes n_mut = 900

- Number of neutral barcodes `n_neutral = 100`
- Ancestral strain growth rate `_a = 1.0`
- Carrying capacity `  = 1.0e10`
- Number of generations `n_gen = 8`
- Initial number of cells `n_init =   / (2^(n_gen)) = 3.90625e7`
- Initial fraction of culture that is ancestor `frac_anc = 0.93`
- Initial fraction of culture that is neutrals `frac_neutral = 0.02`
- Initial fraction of culture that is mutants `frac_mut = 0.04999999999999995`
- Mutant fitness distribution mean `_bc_mean = _a * 1.005 = 1.005`
- Mutant fitness distribution standard deviation to sample growth rates `_bc_std = 0.1`
- Mutant fitness distribution truncation ranges for growth rates `_trunc = [_a .* 0.9999, _a * 1.5] = [0.9999, 1.5]`
- Gaussian noise distribution standard deviation `_lognormal = 0.3`
- Number of unique genotypes `n_geno = Int64(n_mut / 10) = 90`

The variable `n_geno` defines the number of unique genotypes in the simulation. In this simulation, we aimed to have ten unique barcodes on average for each genotype. The actual number of genotypes per barcode was sampled from a multinomial distribution with probability `1 / n_geno` for each genotype.

E. Comparison with `FitSeq2.0`

For a complete comparison, we apply our inference pipeline to a simulated dataset closely following the simulation pipeline from Li, Tarkington, and Sherlock [6]. This simulation explicitly accounts for multiple noise sources captured in the added Gaussian noise in Section D. Here, we list all the steps involved in this explicit simulation, highlighting that the overall results are qualitatively very similar to that of our simple logistic growth plus added Gaussian noise when computing the relevant quantity of the log frequency ratio.

1. We initialized a population consisting of 1000 unique barcodes—50 neutral barcodes and 950 mutant barcodes—where the initial number of cells with barcode i in the culture was sampled from a Gamma distribution as

$$n_o^{(i)} \sim \text{Gamma}(20, 0.2) \tag{S108}$$

2. The Malthusian fitness (related but not exactly the relative fitness to be inferred from the data) for non-neutral barcodes was then sampled from a skewed normal distribution of the form

$$\lambda^{(j)} \sim \text{Skew}\mathcal{N}(0, 0.225, 3) \tag{S109}$$

3. To simulate the noisy growth, the number of cells underwent the following steps:

for $t = 2 : T$ **do**

$n_{t,c}^{(i)} = \text{Poisson}(n_{t-1,c}^{(i)} e^{\lambda^{(i)}})$

end for,

where T is the number of generations, and c indexes the growth-dilution cycle. We use $T = 4$ for our simulation.

4. After the T generations, cells were stochastically diluted back for the next growth-dilution cycle as

$$n_{0,c+1}^{(i)} \sim \text{Poisson}\left(\frac{n_{T,c}^{(i)}}{2^T}\right). \tag{S110}$$

   This process was repeated $C = 4$ times for a total of four growth-dilution cycles.

5. To simulate the sampling noise when taking cells that will then be subsequently processed to obtain barcode reads, we sampled $n_{\text{sample},c}^{(i)}$ cells with barcode i as

$$n_{\text{sample},c}^{(i)} \sim \text{Poisson}(L \times N \times f_c^{(i)}), \tag{S111}$$

   where c indexes the growth cycle, N is the total number of unique barcodes, $f_j^{(i)}$ is the fraction of cells in the culture with barcode i at the end of cycle j, i.e.,

$$f_c^{(i)} = \frac{n_c^{(i)}}{\sum_{j=1}^{N} n_c^{(j)}}, \tag{S112}$$

   and L is the expected average number of cells per lineage. We used $L = 500$.

6. To simulate the noise due to PCR amplification, we iterate $c_{\text{PCR}} = 25$ times, stochastically doubling the number of barcodes with a Poisson sample of the form

for $a = 1 : A$ **do**

$n_{\text{PCR},(a+1),c}^{(i)} \sim \text{Poisson}(2n_{\text{PCR},(a),c}^{(i)})$

end for,

where (a) indexes the amplification cycle, A is the total number of cycles, and c indexes the growth-dilution cycle. We use $A = 25$ for our simulation. For $a = 1$, we use the number of cells sampled in Equation S111.

7. To simulate the noise due to the sampling step involved during the sequencing of the amplicon library, the number of reads mapping to barcode i at the end of cycle c used in the inference was sampled as

$$n_{\text{seq},c}^{(i)} \sim \text{Poisson}(R \times N \times f_{\text{PCR},c}^{(i)}), \tag{S113}$$

   where R is the expected number of reads per barcode (R=100 for our simulation), N is the number of total barcodes, and $f_{\text{PCR},c}^{(i)}$ is the fraction of barcodes of type i in the final amplification cycle from step 4.

Figure F(A) and (B) show the simulation's frequency and log frequency ratio trajectories, respectively. Qualitatively, these results are very similar to those shown in Fig. 1 of Li, Tarkington, and Sherlock [6]. As done throughout the main text, Figure F(C) shows examples of the posterior predictive checks for the neutral lineages and a few of the mutant lineages. We can see that our inference pipeline can fit this simulated data for the examples shown. Furthermore, Figure F(D) compares the ground truth fitness values and the inferred parameters for all mutant lineages. Figure F shows the comparison between the inference with the software provided in [6] and our inference pipeline. We note that although the values are highly correlated, the scale of the axis is very different. This scale difference comes from the differences in models used to infer the relative fitness value. While our model assumes an exponential change in the relative frequency of the barcode, Li, Tarkington, and Sherlock [6] works directly with the number of cells rather than the relative frequency, assuming a Malthusian fitness form. In other words, while our model is agnostic to the underlying type of growth mechanism, as long as the condition of exponential change in relative frequency is satisfied, Li, Tarkington, and Sherlock [6] assumes exponential growth of the number of cells, resulting in a discrepancy between the obtained numbers.

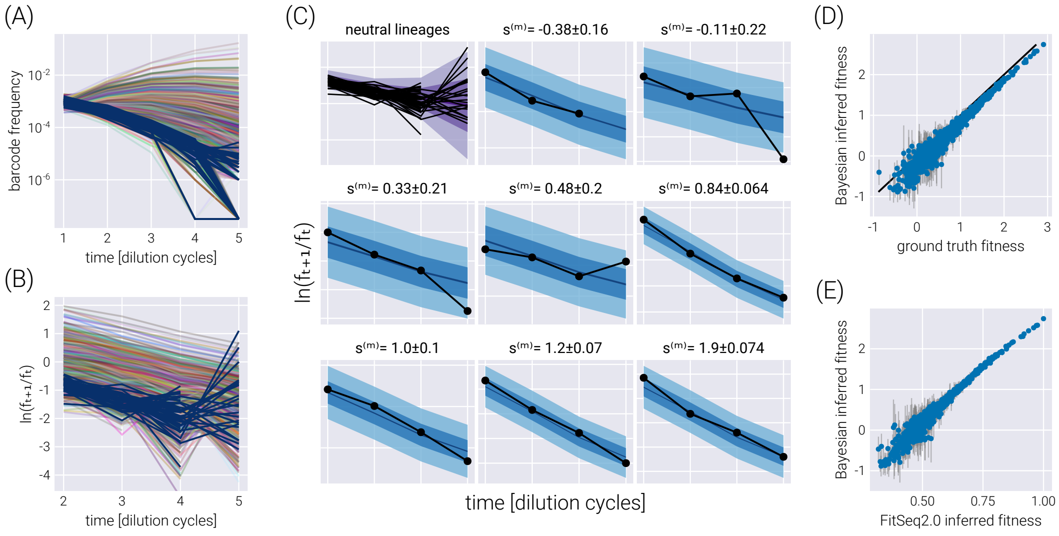

Figure F. FitSeq2.0 simulation comparison.(A) Frequency trajectories that represent the raw data going into the inference. (B) Log frequency ratio between two adjacent time points used by the inference pipeline. Darker lines represent the neutral barcodes. These transformed data are much more noisy than the seemingly smooth frequency trajectories. (C) Examples of the posterior predictive checks for all neutral lineages (upper left panel) and a subset of representative mutant lineages. Shaded regions represent the 95%, 68%, and 5% credible regions for the data. The reported errors above the plot represent the 68% credible region on the mutant relative fitness marginal distribution. (D) Comparison between the ground truth fitness value from the logistic-growth simulation and the inferred fitness value. Gray error bars represent the 68% posterior credible region for the relative fitness values. (E) Comparison between fitness inferences as obtained using `FitSeq2.0` from Li, Tarkington, and Sherlock [6] and our Bayesian method. Vertical gray error bars represent the 68% posterior credible region for the relative fitness values.

F. Reanalyzing Kinsler et al., 2020

The main text of this paper focuses only on simulation-based assessments of the performance of our inference algorithm. This supplementary section shows how the model performs on experimental data. In particular, we focus on a subset of the data generated for [7]. In this paper, the fitness of a set of mutants originally evolved in minimal glucose media was determined in multiple environments using an experimental design described in Section 2.1. Figure G shows the raw barcode frequency trajectories for four of such environments measured in two biological replicates.

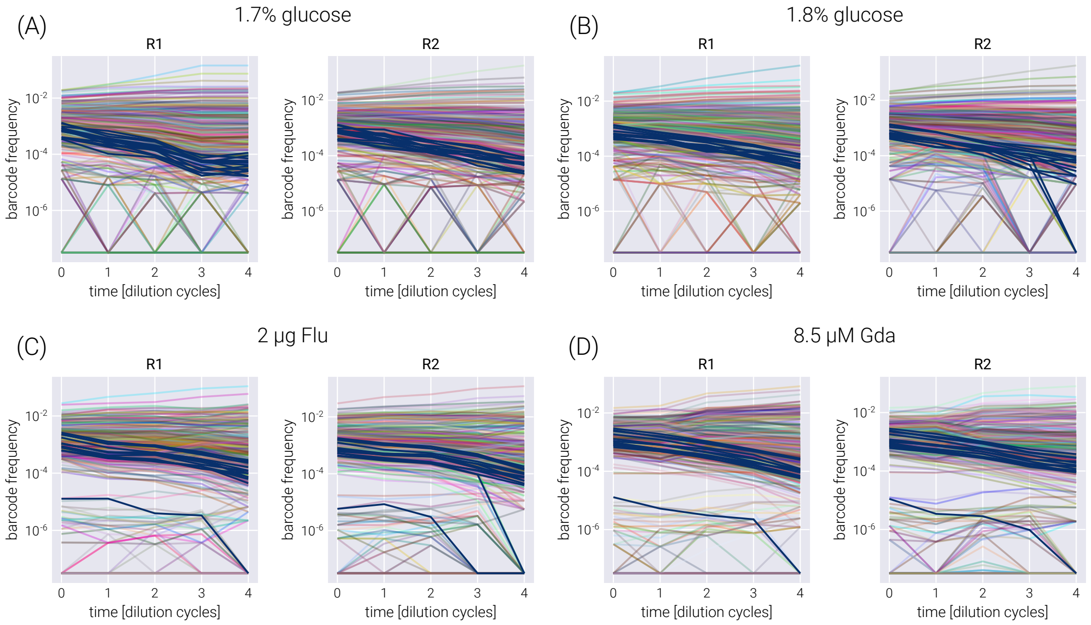

Figure G. Kinsler et al., 2020 sample data. Barcode frequency trajectories in four different environments for mutants evolved in minimal glucose media. Each environment was measured in two biological replicates (R1 and R2 on each panel). For experimental details, see [7].

F.1. Analysis of individual replicates

As a first analysis, we perform inference assuming all replicates are independent. Figure H shows the posterior predictive checks for the neutral lineages (upper left plot on each panel) and some sample mutants for both replicates. We can see that the inference works exceptionally well with the data, capturing the variability. Although the main text inferences were made on logistic growth simulations, as described in Section D, the results in Section E and this section show that our model does not depend on the assumptions underlying such simulations. The only assumption that must be valid is that the changes in the relative frequencies of each lineage follow an exponential function.

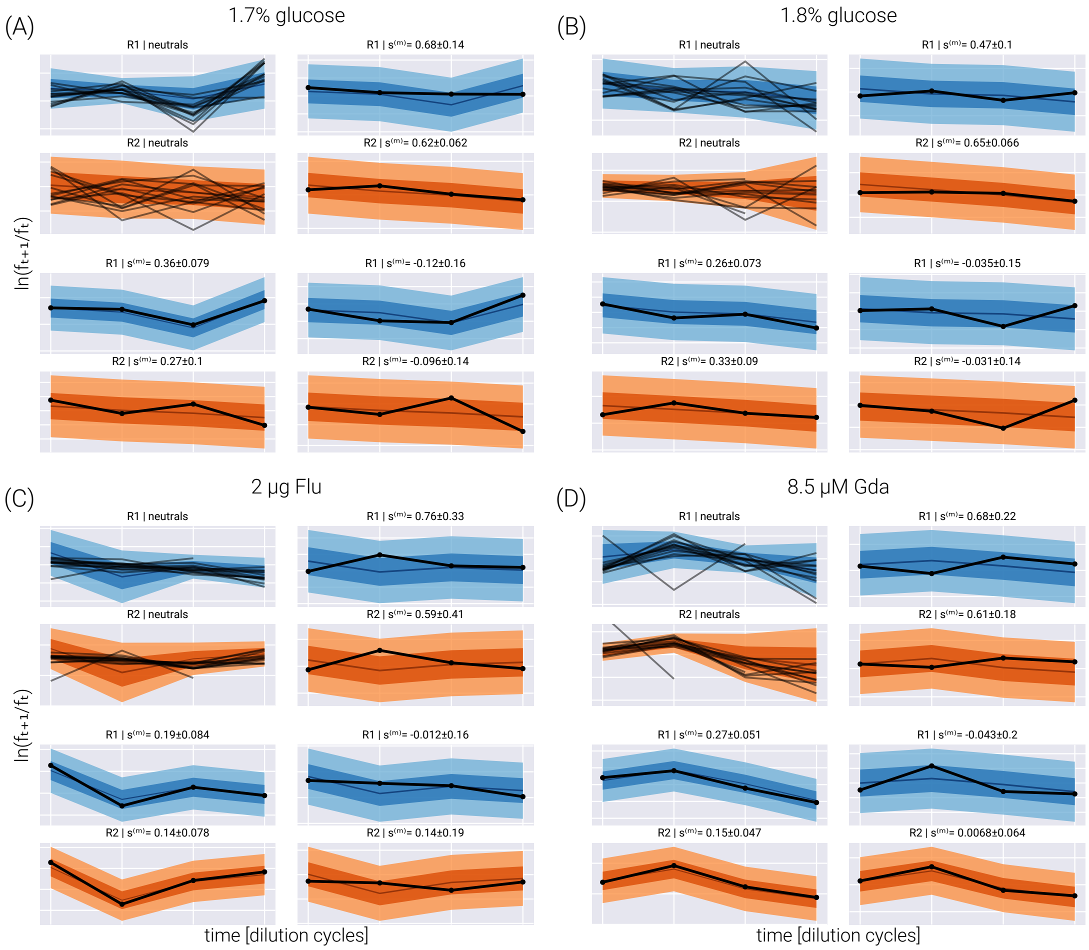

Figure H. Posterior predictive checks for Kinsler et al., 2020 independent replicates. Examples of the posterior predictive checks for all neutral lineages (upper left panels) and a subset of representative mutant lineages for all four environments shown in Figure G. Shaded regions from light to dark represent the 95%, 68%, and 5% credible regions. Listed fitness values for mutant lineages represent the 68% credible region.

Furthermore, we can compare the replicate-to-replicate correlation for these four environments with two biological replicates. The result of this comparison is shown in Figure I. We can see that there is a good degree of correlation between replicates.

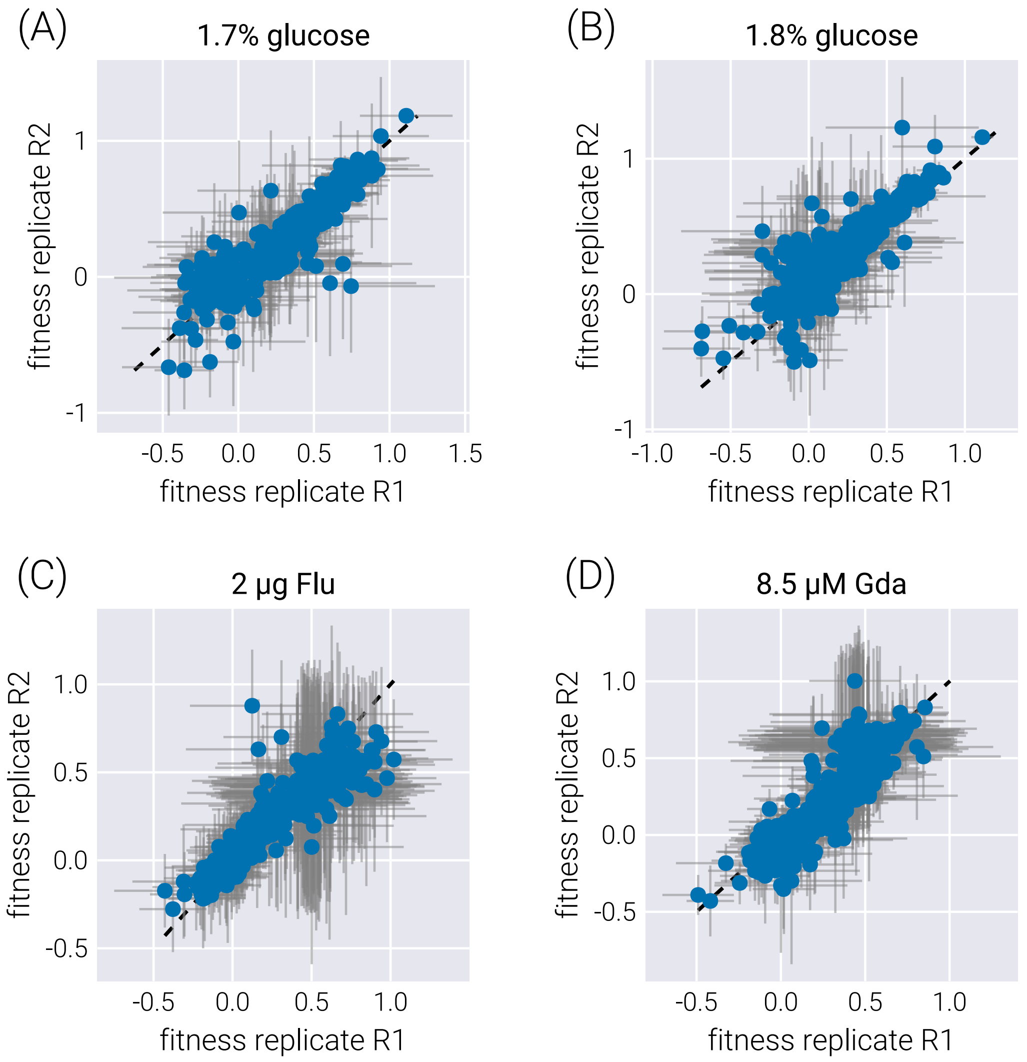

Figure I. Replicate-to-replicate correlation for single-dataset model. Comparison of inferred relative fitness values for mutant strains between experimental replicates when fitting each replicate individually for all environments shown in Figure G. Gray error bars show the 68% credible region for this inference.

Given that we have two biological replicates of the same experiment, we can ask how the difference between each replicate's inferred fitness values compares with the individual estimates' uncertainty. In other words, we want to compare how different the x- and y-axis values in Figure I are with the size of the shown error bars. Figure J compares the distribution of these magnitudes for all four environments. In each plot, the blue line shows the

empirical cumulative distribution function (ECDF) of the distance between the mean fitness estimate for each replicate—computed as $\sqrt{\left(\left\langle s_1^{(m)}\right\rangle - \left\langle s_2^{(m)}\right\rangle\right)^2}$, where $\langle s_i^{(m)}\rangle$ is the mean fitness estimate of mutant m in replicate i—meaning the difference between x- and y-axis in Figure I. The orange and green lines represent the ECDF for each replicate's posterior distribution standard deviation, i.e., the ECDF of the size of the error bars in Figure I. From these plots, we can see that the distribution of standard deviations is shifted to the left compared to the difference between replicates. This implies that the uncertainty in the estimate is generally larger than the difference between replicates one would obtain with a naive point estimate.

F.2. Hierarchical analysis of multiple replicates

As discussed in Section 2.7, most experimental designs—including that of Kinsler, Geiler-Samerotte, and Petrov [7]—include two or more biological replicate measurements. Therefore, a natural way to analyze the data is by jointly inferring the fitness value using a hierarchical model. Figure K shows the posterior predictive checks when fitting a hierarchical model to each of the four environments shown in Figure G. Comparing this figure with Figure H shows that the hierarchical model does not lose the ability to capture the variability in the data compared to fitting each replicate individually. However, as shown in Figure L, the hierarchical model does a better job when comparing the replicate-to-replicate correlation, as expected. To emphasize this last point, Figure M shows both inferences on the same plot—only the mean of the posterior distribution is shown for visual clarity.

We can again compare the magnitude of the difference between replicates' inferred mean fitness with the size of the corresponding error bars, as we did with Figure J. Figure N shows the ECDF for the resulting difference between replicates mean fitness in blue as well as the resulting standard deviation for the inferred hyper-fitness parameter that connects both replicates in orange (see Section 2.7 for description of this hyper-fitness parameter). Here, we see that the standard deviation ECDF (orange line) is shifted more to the right than the mean difference between replicates (blue line).

Even though the difference between replicates is reduced when using a hierarchical model, this does not come at the expense of higher uncertainty in the estimate of the relative fitness. To show this is the case, Figure O compares the reported posterior distribution standard deviations for the relative fitness when inferring each dataset individually (x-axis) with the standard deviation of the hyper-fitness parameter (y-axis). Most of the points fall below the identity line, demonstrating that the uncertainty in the hyper-fitness parameter obtained by utilizing information from both replicates is smaller than the equivalent uncertainty obtained with one replicate at a time. In conclusion, the hierarchical model not only can equally describe the data, as shown in Figure K, but the difference between replicates is significantly decreased, as shown in Figure M, at the same time, the uncertainty in the parameters' estimate is decreased, as shown in Figure O.

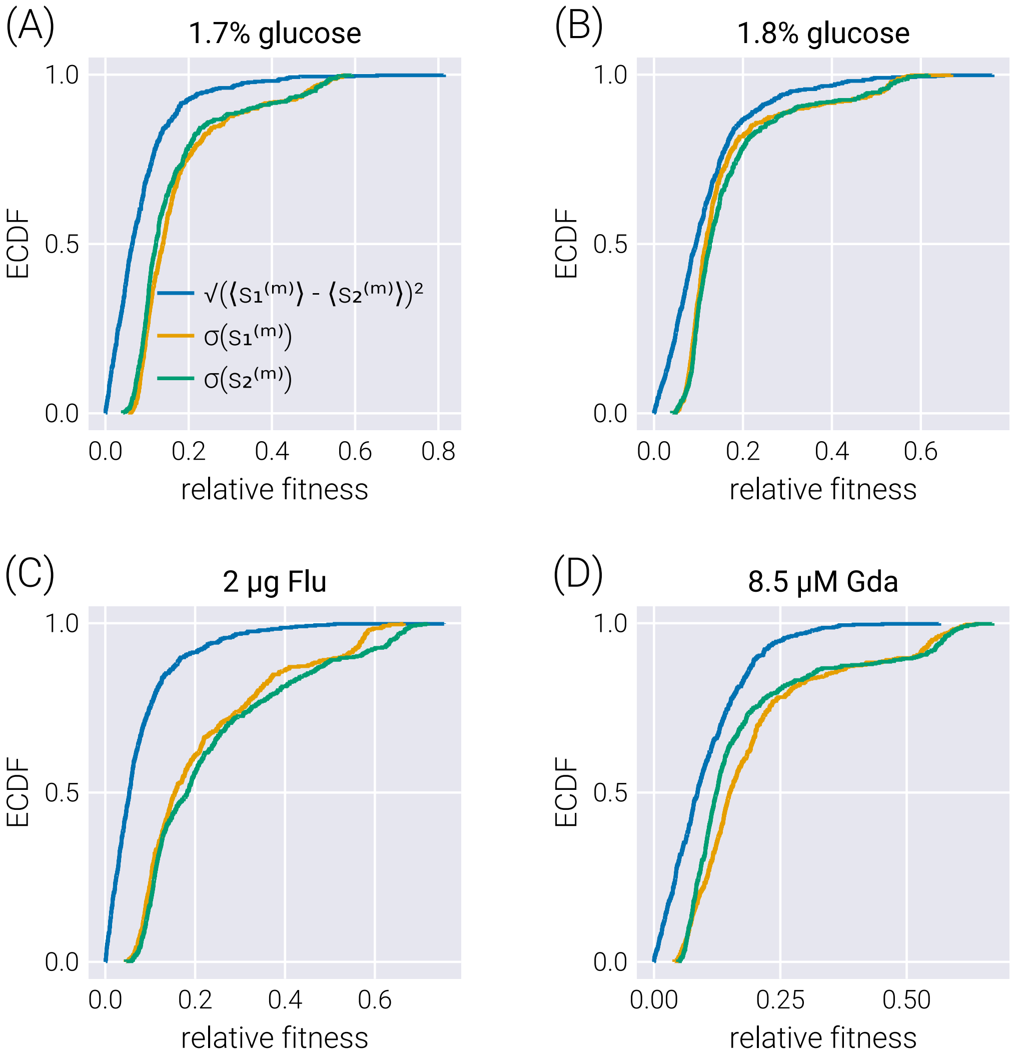

Figure J. Comparing the replicate-to-replicate deviations with within-batch noise estimates for single dataset analysis. Empirical cumulative distribution functions of the distance between the mean fitness estimate for each replicate (blue lines) and each replicate's posterior distribution standard deviation (orange and green line).

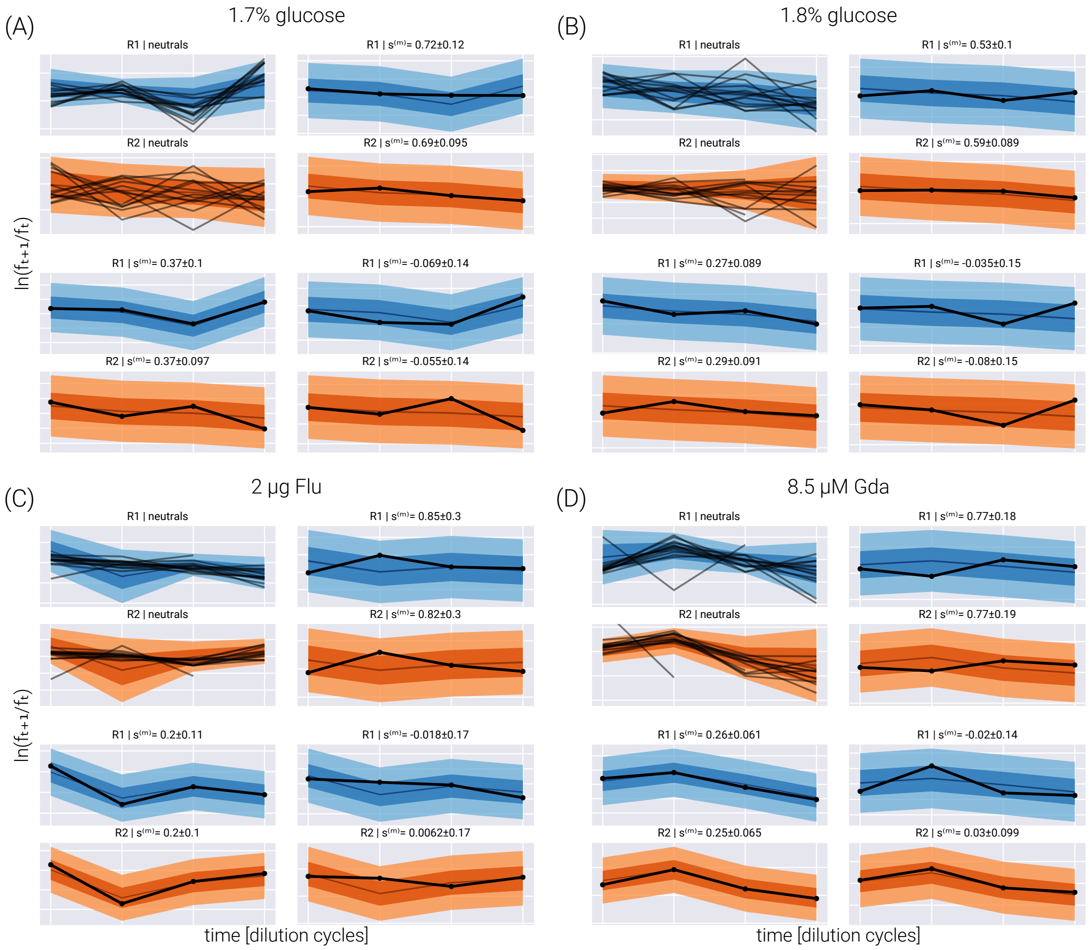

Figure K. Posterior predictive checks for Kinsler et al., 2020 hierarchical model. Examples of the posterior predictive checks for all neutral lineages (upper left panels) and a subset of representative mutant lineages for all four environments shown in Figure G. Shaded regions from light to dark represent the 95%, 68%, and 5% credible regions. Listed fitness values for mutant lineages represent the 68% credible region.

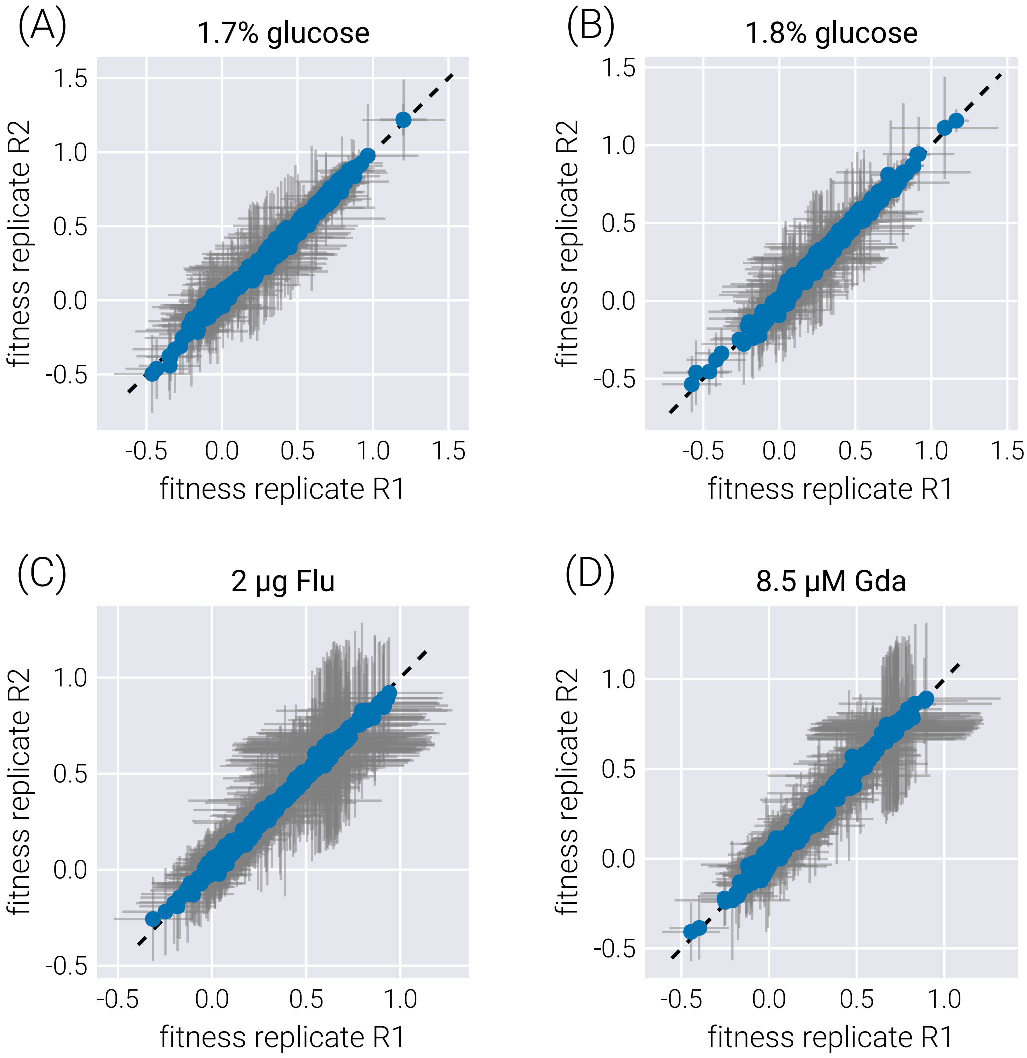

Figure L. Replicate-to-replicate correlation for hierarchical model. Comparison of inferred fitness values between experimental replicates when fitting a hierarchical model on the biological replicates for all environments shown in Figure G. Gray error bars show the 68% credible region for this inference.

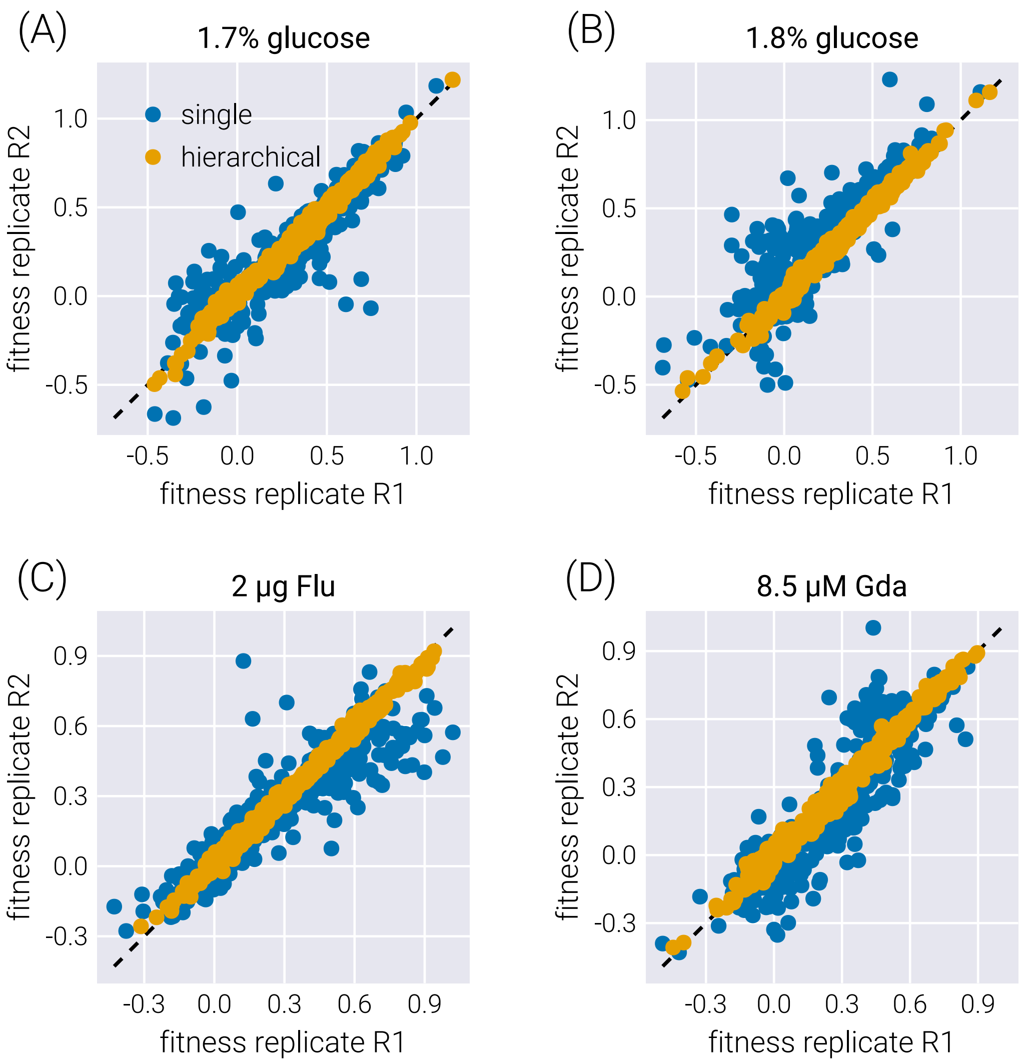

Figure M. Replicate-to-replicate correlation for both methods. Comparison of **mean** inferred fitness values between experimental replicates when fitting a single-dataset model (blue points) vs. a hierarchical model on the biological replicates (orange points) for all environments shown in Figure G. Error bars are not included in the plot for visual clarity

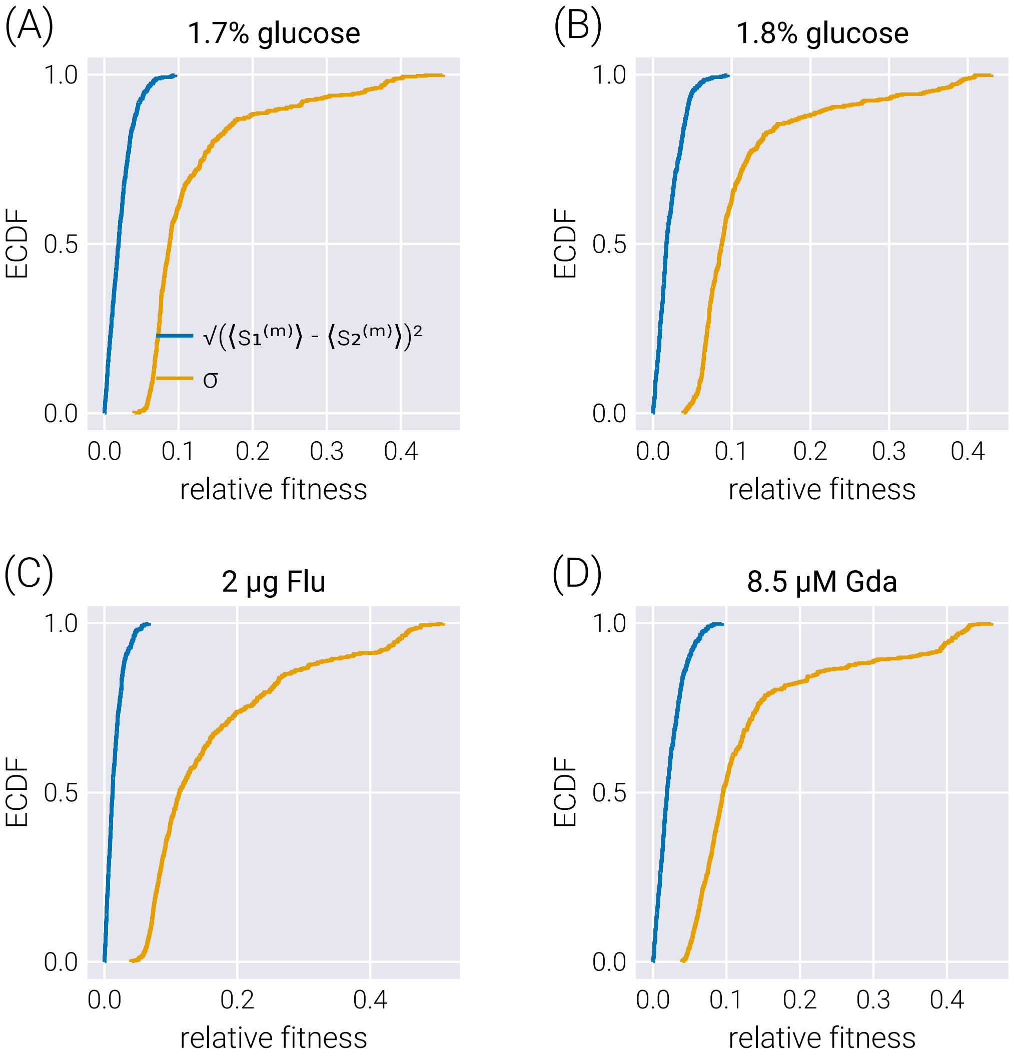

Figure N. Comparing the replicate-to-replicate deviations with within-batch noise estimates for hierarchical model analysis. Empirical cumulative distribution functions of the distance between the mean fitness estimate for each replicate (blue lines) and each replicate's posterior distribution standard deviation (orange and green line).

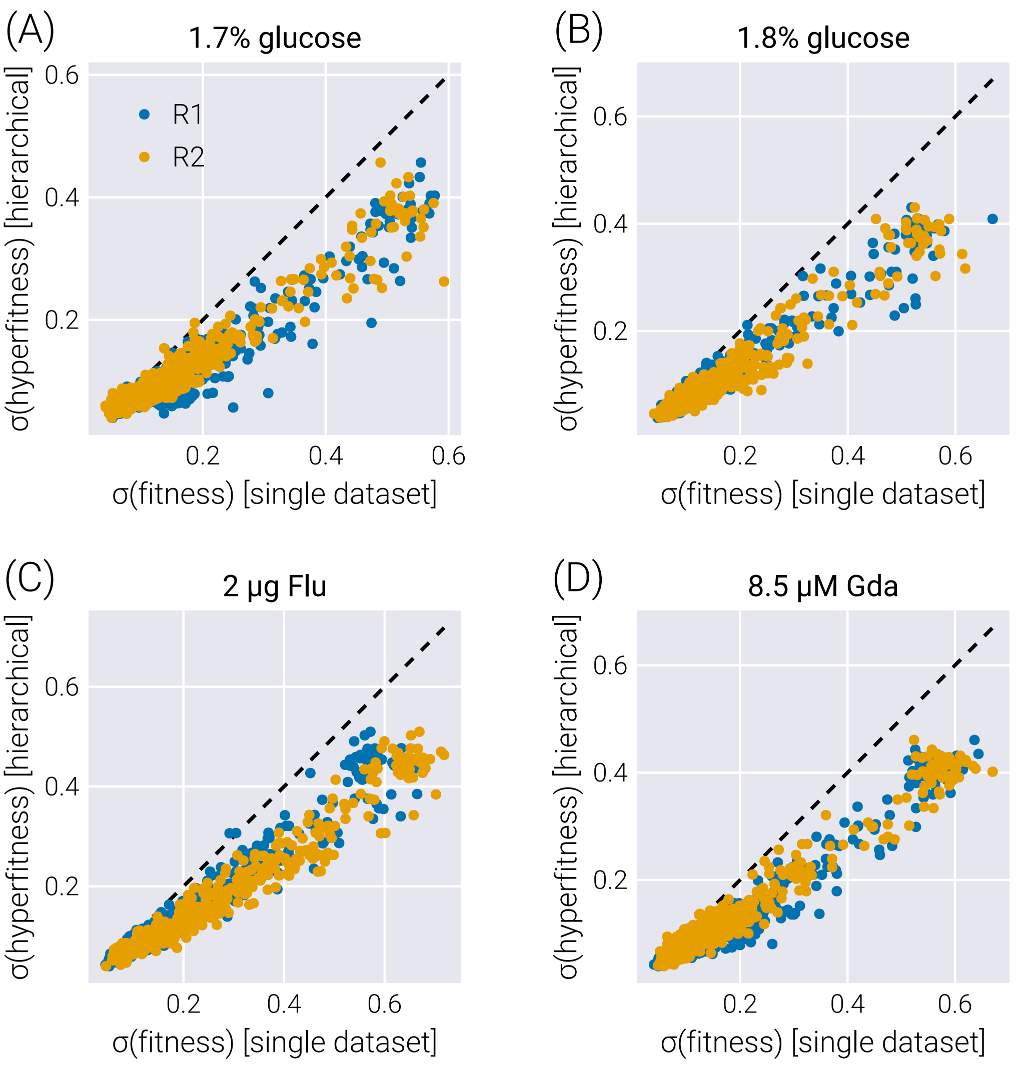

Figure O. Comparing estimates uncertainties between single-dataset and hierarchical model. Comparison of the reported standard deviations for the mutants' relative fitness when inferring the relative fitness using one dataset at a time (x-axis) and the corresponding standard deviation for the mutants' hyper-fitness parameter when utilizing both replicates simultaneously via a hierarchical model (y-axis). Dashed line shows identity line.

G. Computation time scaling

The biggest advantage of the variational inference approach presented in this work is the computation speedup compared to MCMC-based Bayesian approaches. This allows for applying our principled Bayesian pipeline to datasets with many unique barcodes or multiple datasets analyzed hierarchically. Figure P shows a linear scaling in the amount of time taken for the ADVI algorithm to take 7,500 steps for a varying number of unique barcodes. This figure shows that the time to fit larger models increases linearly with the number of barcodes.

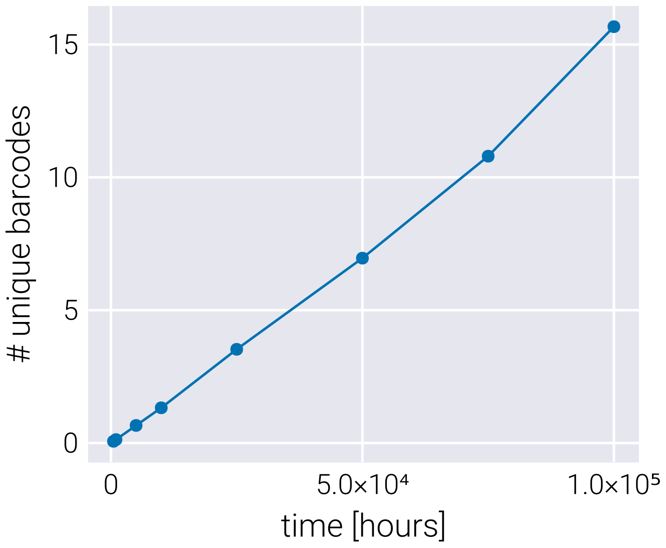

Figure P. Scaling of computation time as a function of number of unique barcodes. Fitting a single-dataset model for 500 to 100,000 unique barcodes by taking 7,500 steps.

Reference

1. Betancourt M. A Conceptual Introduction to Hamiltonian Monte Carlo. ArXiv. 2017
2. Kingma DP and Welling M. Auto-Encoding Variational Bayes. 2014 May 1. Available from: http://arxiv.org/abs/1312.6114 [Accessed on: 2022 Nov 21]. preprint
3. Kucukelbir A, Tran D, Ranganath R, Gelman A, and Blei DM. Automatic Differentiation Variational Inference. 2016 Mar 2. preprint
4. Betancourt MJ and Girolami M. Hamiltonian Monte Carlo for Hierarchical Models. 2013 Dec 3. Available from: http://arxiv.org/abs/1312.0906 [Accessed on: 2023 Jul 20]. preprint
5. Gelman A, Simpson D, and Betancourt M. The Prior Can Often Only Be Understood in the Context of the Likelihood. Entropy. 2017 Oct 19; 19:555

6. Li F, Tarkington J, and Sherlock G. Fit-Seq2.0: An Improved Software for High-Throughput Fitness Measurements Using Pooled Competition Assays. Journal of Molecular Evolution. 2023 Mar 6

7. Kinsler G, Geiler-Samerotte K, and Petrov DA. Fitness Variation across Subtle Environmental Perturbations Reveals Local Modularity and Global Pleiotropy of Adaptation. eLife. 2020 Dec 2; 9:1–52
