# Supplementary material for: Bayesian inference of relative fitness on high-throughput pooled competition assays
Source: PLoS Comput Biol. 2024 Mar 15;20(3):e1011937. doi: 10.1371/journal.pcbi.1011937 (PMC10971673; doi:10.1371/journal.pcbi.1011937)
Supplement: Attachment — Submitted filename: ploscompbio_review_11122023.pdf [file pcbi.1011937.s002.pdf]

In this work, Razo-Mejia, Mani, and Petrov present a Bayesian inference pipeline for estimating relative fitnesses in barcoded microbial evolution experiments. The work contributes a careful analysis of some of the sources of uncertainty in these experiments, and presents a principled statistical model that incorporates these uncertainties. The use of newer approximate inference techniques (ADVI) to make the computations tractable is also a technical advance. The paper is technically sound and the exposition is clear.

However, I think that there are some additional important demonstrations of the feasibility and utility of the method, that the authors should perform.

Primary concern: There is no inference on real data in the paper. The inference was performed on simulations with 1000 focal barcodes, whereas real experiments of this type usually have 500000 barcodes, and sometimes even more. Given that this is a method specifically for inferring fitnesses for this type of experiment, I think it's important to demonstrate that this computation is feasible. (There was no discussion of computational time and its scaling with barcode number.)

Other major comments:

As the authors note, there are existing methods to infer fitnesses in these experiments. The authors' main claim is that their method handles experimental noise in a more principled way.

1. Related to this claim, it would greatly strengthen the paper to have some information about the scale of variability observed in the experiments between replicate flasks in one assay, and between assays, vs. the modeled within-batch variability. While it is understood that there are 'real' biological batch effects, if the within-batch errors estimated here are far smaller than what is experimentally achievable, they may be of questionable utility.

2. It would be valuable to have some discussion of the ways in which the experiments are not fully captured by the model. (See, e.g., minor comment 1 below) Absent evidence from experimental data, it is hard to know whether the error model used here actually does capture the most important sources of experimental noise.

I would be far less concerned about the details of the error estimation were it not for the fact that, given other options for inferring the fitnesses themselves, this is the main claim to novelty of the paper.

Additional minor comments:

1. Related to the model assumptions, microbes do not grow purely logistically in these conditions, but rather have a lag phase (and yeast also go through diauxic shift). The rate at which different genotypes exit lag phase can contribute to fitness, and can also be affected by the time spent in stationary phase prior to dilution (one of the sources of experimental variation between assays). It would be useful to comment on the expected magnitude of these effects, and others that are not modeled, relative to sources of error that are in the model.
2. One of the main functions of careful quantitative analysis like this is to know when to be surprised. It would be useful to discuss whether it is possible to use this model to detect, for example, interesting deviations from the expectation of logistic growth (frequency dependent fitness, etc.)
3. For the inference of fitnesses when multiple barcodes are assigned to the same genotype, could you clarify whether it is necessary for the method to know which barcodes go with which genotypes in advance? (There is also a more complex inference problem that would

involve inferring which barcodes have the same fitness as one another directly from the data, and grouping those)

4. This reader did not find the discussion of frequentist vs. bayesian confidence interval estimation (paragraph at line 244) useful. On a related note, the authors might consider re-wording the final sentence of the abstract.
