# Supplementary material for: Bayesian inference of relative fitness on high-throughput pooled competition assays
Source: PLoS Comput Biol. 2024 Mar 15;20(3):e1011937. doi: 10.1371/journal.pcbi.1011937 (PMC10971673; doi:10.1371/journal.pcbi.1011937)
Supplement: Attachment — Submitted filename: ploscompbio_reviews.pdf [file pcbi.1011937.s003.pdf]

PLoS Comp. Bio. reviews for Bayesian inference of relative fitness on high-throughput pooled competition assays

Manuel Razo-Mejia[1,*], Madhav Mani[3,4], and Dmitri Petrov[1,2,5]

[1] *Department of Biology, Stanford University*
[2] *Stanford Cancer Institute, Stanford University School of Medicine*
[3] *NSF-Simons Center for Quantitative Biology, Northwestern University*
[4] *Department of Engineering Sciences and Applied Mathematics, Northwestern University*
[5] *Chan Zuckerberg Biohub*

Dear Editors,

We want to express our sincere gratitude for the opportunity to submit our revised manuscript, titled "Bayesian inference of relative fitness on high-throughput pooled competition assays," and the accompanying responses to the reviewers' comments. We deeply appreciate the time and effort invested by the reviewers in evaluating our work.

The constructive feedback provided was helpful, not only in enhancing the overall quality of the manuscript but also in contributing significantly to our understanding and perspective on the subject. Each comment was carefully considered and addressed, which we believe has substantially improved the manuscript.

We are particularly grateful for the reviewers' comments into the lack of application of our pipeline to experimental data, which prompted a thorough validation of the overall approach visible to all readers. This feature has enriched the study, making the findings more robust and compelling.

We are hopeful that these revisions meet the high standards of PLOS Computational Biology and contribute meaningfully to the field. We are also looking forward to the possibility of our research being shared within the scientific community through your journal.

Thank you once again for facilitating such a rigorous and constructive peer-review process. Please convey our gratitude to the reviewers for their valuable contributions to the refinement of this manuscript.

Yours sincerely,

The authors

Reviewer 1

> In this work, Razo-Mejia, Mani, and Petrov present a Bayesian inference pipeline for estimating relative fitnesses in barcoded microbial evolution experiments. The work contributes a careful analysis of some of the sources of uncertainty in these experiments, and presents a principled statistical model that incorporates these uncertainties. The use of newer approximate inference techniques (ADVI) to make the computations tractable is also a technical advance. The paper is technically sound and the exposition is clear.
> However, I think that there are some additional important demonstrations of the feasibility and utility of the method, that the authors should perform.
> Primary concern: There is no inference on real data in the paper. The inference was performed on simulations with 1000 focal barcodes, whereas real experiments of this type usually have 500000 barcodes, and sometimes even more. Given that this is a method specifically for inferring fitnesses for this type of experiment, I think it's important to demonstrate that this computation is feasible. (There was no discussion of computational time and its scaling with barcode number.)

We thank the reviewer for highlighting this concern. As the reviewer will be able to see in the following responses to the other comments and the new section S1.6 in the supplementary materials, we have applied our analysis pipeline to real datasets. Furthermore, we found the reviewer's suggestion on analyzing the scaling of the computation time as a function of the size of the dataset very relevant. For this, we fit models on simulated datasets for increasing numbers of unique barcodes. As detailed in a new short supplementary material section (section S1.7) and reproduced here in Figure 1, the computation time scales linearly with the number of unique barcodes in the dataset. Although taking roughly 15 hours to fit a dataset with 100,000 unique barcodes—almost 700,000 parameters in total—might seem relatively slow, this amount of parameters would be unimaginably slow with traditional MCMC-based approaches. Thus, our variational approximation makes the principled analysis of large datasets possible.

> Other major comments: As the authors note, there are existing methods to infer fitnesses in these experiments. The authors' main claim is that their method handles experimental noise in a more principled way.
>
> 1. Related to this claim, it would greatly strengthen the paper to have some information about the scale of variability observed in the experiments between replicate flasks in one assay, and between assays, vs. the modeled within-batch variability. While it is understood that there are 'real' biological batch effects, if the within-batch errors estimated here are far smaller than what is experimentally achievable, they may be of questionable utility.

We thank the reviewer for this excellent suggestion. In our new supplementary section,

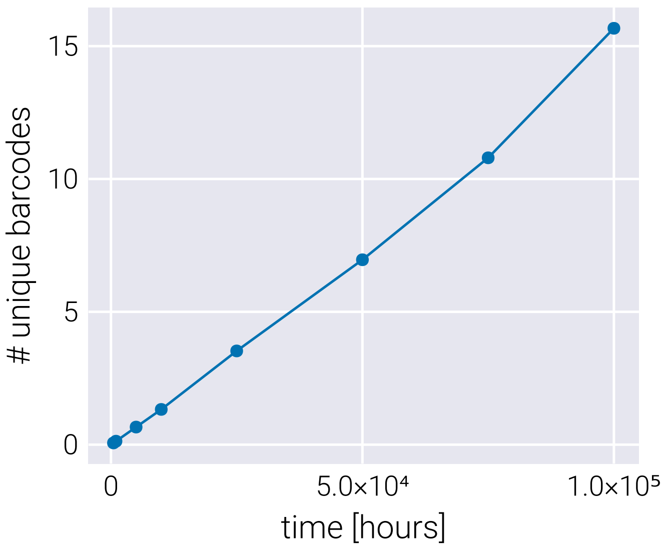

Figure 1. Scaling of computation time as a function of number of unique barcodes. Fitting a single-dataset model for 500 to 100,000 unique barcodes by taking 7,500 steps.

where we re-analyze part of the dataset generated by Kinsler et al. [1], we included an analysis based on this comment. Briefly, we analyzed barcode trajectories for ≈ 500 mutants in four different environments measured in two biological replicates in two different ways: first, assuming each replicate is independent of the other, and then utilizing the hierarchical model for experimental replicates presented in the main text. For the single dataset analysis, we can see that there is a good degree of correlation between the biological replicates, as reproduced here in Figure 2.

However, as we mention in the new supplementary section, inspired by the reviewer's excellent comment:

> Given that we have two biological replicates of the same experiment, we can ask how the difference between each replicate's inferred fitness values compares with the individual estimates' uncertainty. In other words, we want to compare how different the x- and y-axis values in Figure S9 (Figure 2 in this document) are with the size of the shown error bars. Figure S10 (Figure 3 in this document) compares the distribution of these magnitudes for all four environments. In each plot, the blue line shows the empirical cumulative distribution function (ECDF) of the distance between the mean fitness estimate for each replicate—computed as $\sqrt{\left(\left\langle s_1^{(m)}\right\rangle - \left\langle s_2^{(m)}\right\rangle\right)^2}$, where $\langle s_i^{(m)}\rangle$ is the mean fitness estimate of mutant m in replicate i—meaning the difference between x- and y-axis in Figure S9 (Figure 2). The orange and green lines represent the ECDF for each replicate's posterior distribution standard deviation, i.e., the ECDF of the size of the error bars in Figure S9 (Figure 2). From these plots, we can see that

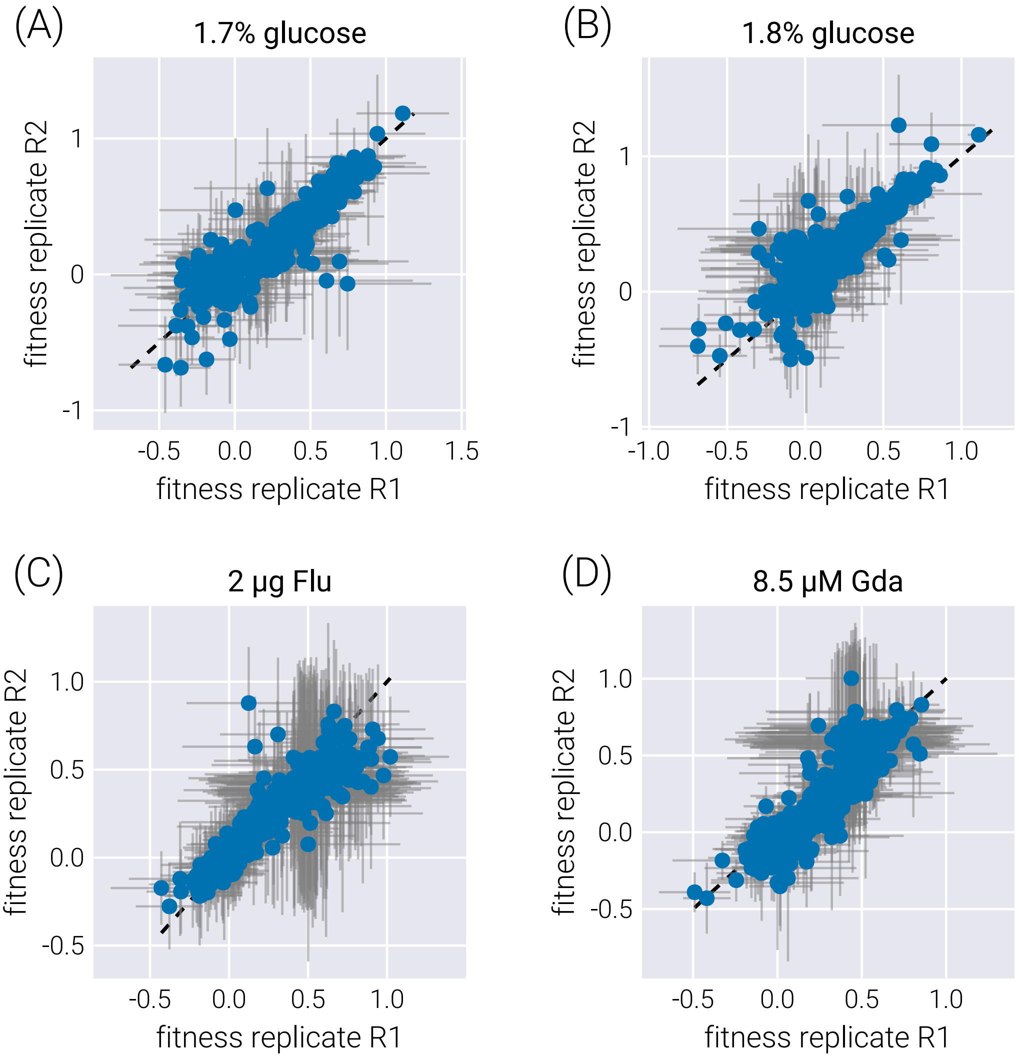

Figure 2. Replicate-to-replicate correlation for single-dataset model. Comparison of inferred relative fitness values for mutant strains between experimental replicates when fitting each replicate individually for four environments from the Kinsler et al. [1] dataset. Gray error bars show the 68% credible region for this inference.

the distribution of standard deviations is shifted to the left compared to the difference between replicates. This implies that the uncertainty in the estimate is generally larger than the difference between replicates one would obtain with a naive point estimate.

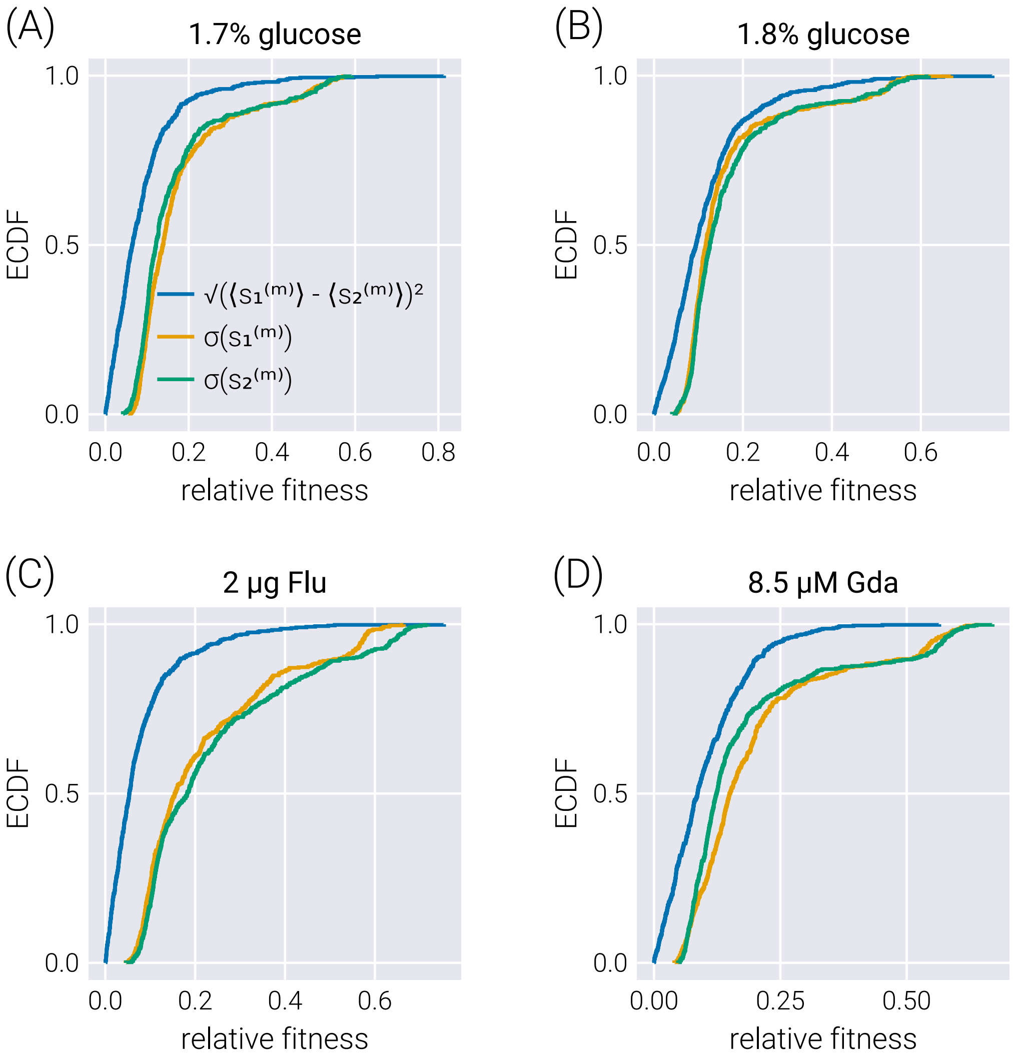

Figure 3. Comparing the replicate-to-replicate deviations with within-batch noise estimates for single dataset analysis. Empirical cumulative distribution functions of the distance between the mean fitness estimate for each replicate (blue lines) and each replicate's posterior distribution standard deviation (orange and green line).

Furthermore, in the supplementary section, we demonstrate that inferring relative fitness via a hierarchical model that utilizes both replicates simultaneously decreases the fitness difference between biological replicates. To illustrate this point, Figure 4 shows the mean (error bars are not shown for visual clarity) fitness for each replicate under both models.

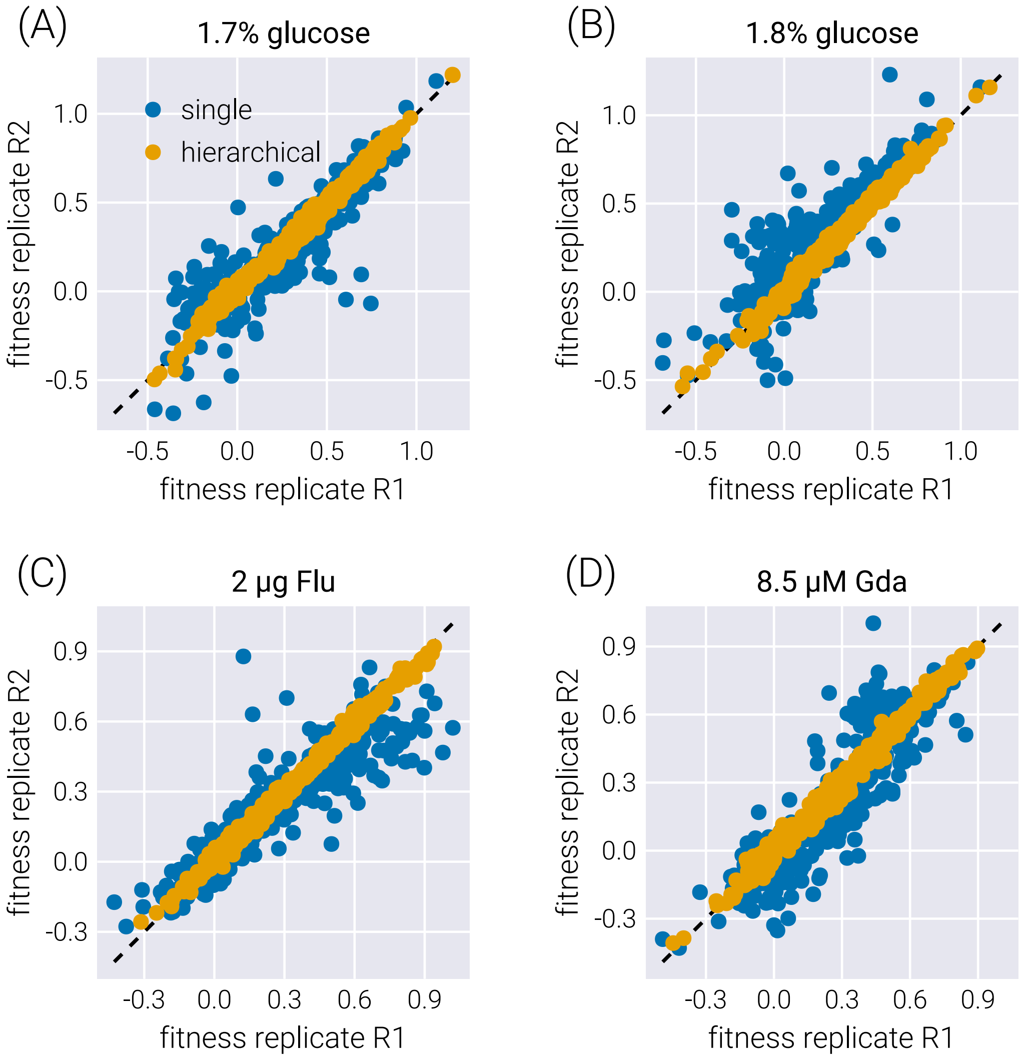

Figure 4. Replicate-to-replicate correlation for both methods. Comparison of **mean** inferred fitness values between experimental replicates when fitting a single-dataset model (blue points) vs. a hierarchical model on the biological replicates (orange points). Error bars are not included in the plot for visual clarity

However, having a higher agreement between biological replicates when fitting a hierarchical model does not come at the cost of a larger uncertainty in the parameter estimate. The opposite is true where the hierarchical model uncertainty on the inferred relative fitness, as characterized by the posterior distribution's standard deviation, is generally smaller than the corresponding standard deviation of the single-dataset inference. This is clearly shown in Figure 5, where we compare the standard deviations for each of the single fits ($x-$axis) with the corresponding standard deviation of the hierarchical model hyper-parameter ($y-$axis). Since most points fall below the identity line, fitting the hierarchical model that uses both replicates simultaneously decreases the uncertainty.

2. It would be valuable to have some discussion of the ways in which the experiments are not fully captured by the model. (See, e.g., minor comment 1 below) Absent evidence from experimental data, it is hard to know whether the error model used here actually does capture the most important sources of experimental noise. I would be far less concerned about the details of the error estimation were it not for the fact that, given other options for inferring the fitnesses themselves, this is the main claim to novelty of the paper.

We thank the reviewer again for this constructive comment on the shortcomings of the paper. As mentioned before the new supplementary section goes into a deep analysis of part of the dataset generated by Kinsler et al. [1]. In there, we show that our model can capture the variability from this real-world experiment, even for cases in which previous work in Li et al. [2] showed that some of the strains' fitness advantage resulted from a shorter lag phase at the beginning of the growth cycle.

Furthermore, in the discussion, we added the following text when listing the points and assumptions behind our inference model:

> This experimental design constraint facilitates the inference of the population mean fitness since most of the culture—the unlabeled reference genotype—is not tracked. Fourth, the fitness model in Equation 3 does not assume any functional form for the cellular growth dynamics. This description of the changes in the relative frequency of the genotypes can approximate the dynamics for multiple growth forms. For example, in Seciton S1.5, in the supplementary materials, we apply our inference pipeline to simulations like those presented in[3] with non-logistic growth models. Moreover, Section S1.6, in the supplementary materials, reanalyzes experimental data from[1], where previous work showed that the fitness advantage from some of the mutants results from a shorter lag phase at the beginning of the growth cycle[2]. Thus, our method's inferred relative fitness is a coarse-grained quantity that integrates the differences in fitness across the entire growth cycle as long as frequencies and frequency changes are small enough to be approximated by Equation 3. Finally, the presented statistical model assumes that relative fitness is solely a constant of the environment and the genotype.

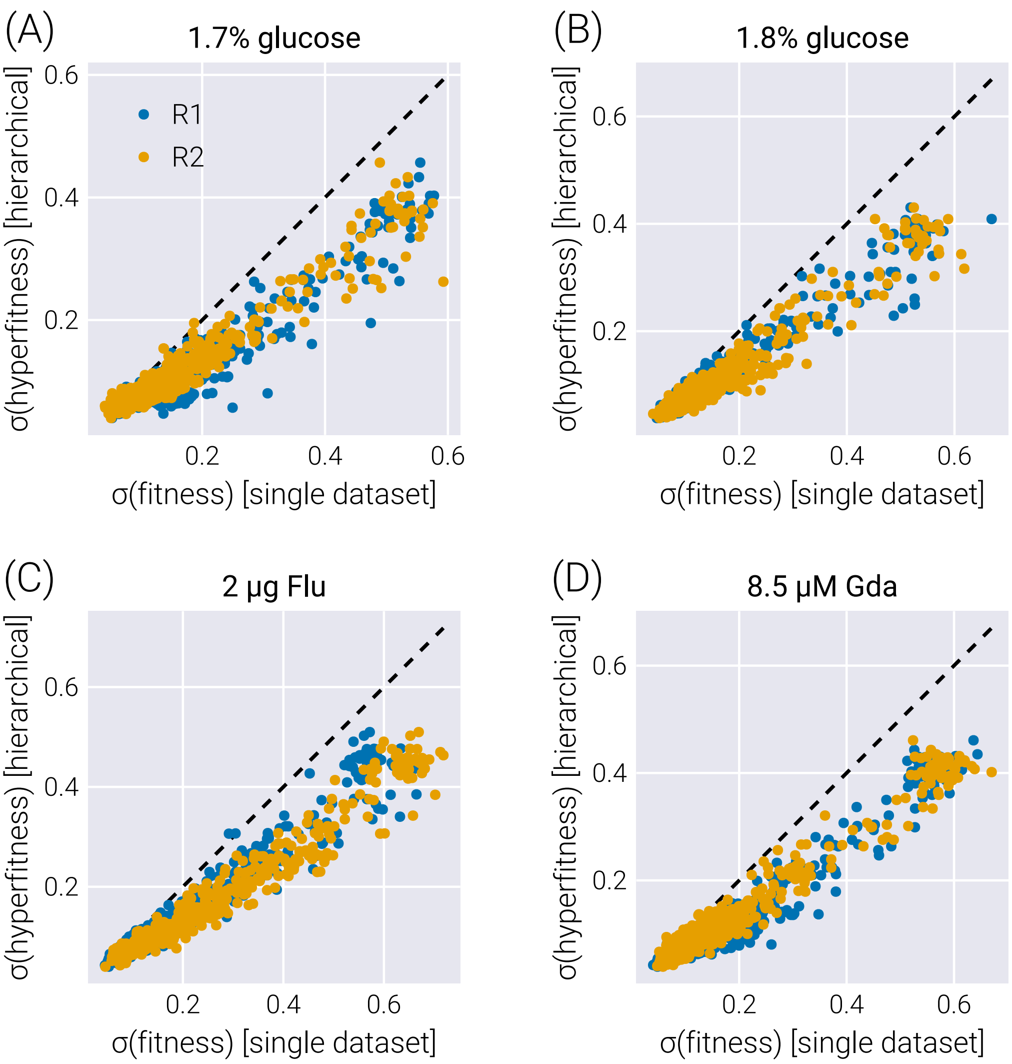

Figure 5. Comparing estimates uncertainties between single-dataset and hierarchical model. Comparison of the reported standard deviations for the mutants' relative fitness when inferring the relative fitness using one dataset at a time (x-axis) and the corresponding standard deviation for the mutants' hyper-fitness parameter when utilizing both replicates simultaneously via a hierarchical model (y-axis). Dashed line shows identity line.

Future directions of this work could extend the fitness model to properly analyze data with time-varying or frequency-dependent fitness values.

Additional minor comments:

1. Related to the model assumptions, microbes do not grow purely logistically in these conditions, but rather have a lag phase (and yeast also go through diauxic shift). The rate at which different genotypes exit lag phase can contribute to fitness, and can also be affected by the time spent in stationary phase prior to dilution (one of the sources of experimental variation between assays). It would be useful to comment on the expected magnitude of these effects, and others that are not modeled, relative to sources of error that are in the model.

We thank the reviewer for this interesting suggestion. We agree that factors such as the length of the lag phase or diauxic shifts can influence the relative fitness of different strains. As a matter of fact, previous work from the lab explored the fitness advantages gained as a function of the phase in the growth cycle[2]. One of the conclusions from this work was that some of the adaptive mutants isolated in that specific experiment gained their fitness advantage from a shorter lag phase at the beginning of the growth cycle.

However, the model presented here does not depend on any assumption related to logistic growth. The assumptions behind the functional form of the frequency change between time points (Eq. 3 in the main text)

$$f_{t+1}^{(b)} = f_t^{(b)} \mathrm{e}^{(s^{(b)} - \bar{s}_t)\tau}, \tag{1}$$

only assumes that the frequency of barcode b, $f_t^{(b)} \ll 1$. One can think of Equation 1 as an approximate solution to the classic result in population genetics for the change in frequency of an allele due to selection only, which takes the form

$$\frac{df^{(b)}}{dt} = s^{(b)} f^{(b)} (1 - f^{(b)}). \tag{2}$$

For the case where $f^{(b)} \ll 1$, the term $1 - f^{(b)} \approx 1$, giving an exponential form for the solution. In a sense, Equation 1 coarse grains the entire growth dynamics into a simple exponential form. As long as the frequency changes between cycles are approximately exponential, our model can determine an "integrated" change in fitness throughout the entire growth cycle. Therefore, The noise sources included in our model are independent of the underlying cellular growth dynamics. To emphasize this point, our discussion now reads:

> Third, the models presented in this paper require the existence of a labeled subpopulation of barcoded reference strains. These barcodes help determine the fitness baseline, as every fitness is quantified with respect to this reference genotype. This experimental design constraint facilitates the inference of the population mean fitness since most of the culture—the unlabeled reference genotype—is

not tracked. Fourth, the fitness model in Equation 3 does not assume any functional form for the cellular growth dynamics. This description of the changes in the relative frequency of the genotypes can approximate the dynamics for multiple growth forms. For example, in Section S1.5, in the supplementary materials, we apply our inference pipeline to simulations like those presented in[3] with non-logistic growth models. Moreover, Section S1.6, in the supplementary materials, reanalyzes experimental data from[1], where previous work showed that the fitness advantage from some of the mutants results from a shorter lag phase at the beginning of the growth cycle[2]. Thus, our method's inferred relative fitness is a coarse-grained quantity that integrates the differences in fitness across the entire growth cycle as long as frequencies and frequency changes are small enough to be approximated by Equation 3. Finally, the presented statistical model assumes that relative fitness is solely a constant of the environment and the genotype. Future directions of this work could extend the fitness model to properly analyze data with time-varying or frequency-dependent fitness values.

2. One of the main functions of careful quantitative analysis like this is to know when to be surprised. It would be useful to discuss whether it is possible to use this model to detect, for example, interesting deviations from the expectation of logistic growth (frequency dependent fitness, etc.)

We thank the reviewer for this intriguing suggestion. Unfortunately, our model cannot comment on such a proposal directly. As previously mentioned, our fitness model does not depend on any assumptions related to logistic growth. All the model assumes is the change in frequency as described in Equation 1. However, the model does assume a constant fitness throughout the growth-dilution cycles. Therefore, our model cannot handle any frequency dependence or time-varying effects. We agree that these time-varying fitness effects are important open problems in the field that have not been fully addressed. In the discussion, we mention the necessity of extending these inference models to consider those biologically relevant cases as interesting future extensions of this work.

3. For the inference of fitnesses when multiple barcodes are assigned to the same genotype, could you clarify whether it is necessary for the method to know which barcodes go with which genotypes in advance? (There is also a more complex inference problem that would involve inferring which barcodes have the same fitness as one another directly from the data, and grouping those)

We thank the reviewer for this suggestion. We agree that having an algorithm that does not need to know the genotype identity of the barcode to group it with other barcodes would be helpful in our approach. However, this would involve clustering barcodes based on their posterior distribution fitness result, which is outside the scope of the present work. To make this distinction clear, the introduction of the section in which the hierarchical model is presented now reads:

> Hierarchical models can also capture another experimental design in which multiple barcodes map to the same or an equivalent genotype. As we will show, this many-to-one mapping can improve the inference compared to the extreme cases of inferring the fitness of each barcode independently or pooling the data of all barcodes mapping to a single genotype. As schematized in Figure 6(A), a small modification of the base model allows us to map the structure of our original model to that of a hierarchical model with a fitness hyperparameter vector $\underline{\theta}^G$, where G is the number of genotypes in the dataset. This analysis pipeline assumes a known barcode-to-genotype mapping to assign each barcode to the corresponding hyper-fitness parameter uniquely.

Nevertheless, we believe that having a full posterior distribution for the relative fitness can help researchers address this question. This is because one can make probabilistic statements, such as computing the probability of two or more barcodes having overlapping fitness values. In other words, if we have a reason to believe there is a small set of genotypes with sufficiently different relative fitness values, we could imagine using the full posterior distribution rather than a single point estimate to make a more informed decision of whether two barcodes belong to the same genotype.

4. This reader did not find the discussion of frequentist vs. bayesian confidence interval estimation (paragraph at line 244) useful. On a related note, the authors might consider re-wording the final sentence of the abstract.

We thank the reviewer for highlighting this point. As the other reviewers agree with the comment, we have removed the extensive discussion. That section now reads:

> The main advantage of our method is the natural interpretability of these credible regions where an $X\%$ credible region indeed captures the region of parameter space where we expect with $X\%$ probability the actual value of the parameter lies given our statistical model, our prior information, and the observed experimental data. Bayesian methods avoid common misconceptions associated with the construction of frequentists confidence intervals[4].

Reviewer 2

By tracking lineages frequency via DNA barcodes in competitive cultures, it is possible to measure microbial fitness and phenotypic diversity on a large scale. However, many biological and non-biological noises will have a significant impact on the relationship between barcodes and phenotypes (fitness). Accordingly, the authors of this manuscript developed a Bayesian model-based pipeline that takes into account some of these uncertainties in the experimental setup described above. Additionally, this model was applied to analyze simulated multi-environment and replicate (by batches, or barcodes of the same genotype) experiments. As a whole, the manuscript has a solid theoretical basis and should be of general interest to the field. I believe that additional work is necessary before it can be published. My concerns are listed below.

MAJOR

1. The manuscript contains a little bit too many technical details, so that readers might become distracted from the main logic flow. As an example, I do not believe it is necessary to comment on frequentist confidence intervals versus Bayesian credible regions starting at line 247. However, I lack a strong statistical background to provide a more subjective or comprehensive assessment or to say that all the technical details are trivial/unnecessary. Other reviewers with expertise in that area may be able to provide better suggestions. Nonetheless, moving some details to supplementary to better emphasize the main logic seems reasonable.

We thank the reviewer for highlighting the excessive level of detail in that section of the paper. We agree that in our attempt to make the paper as thorough as possible, we could overwhelm the reader. To shorten the discussion without missing emphasis on what we believe is the crucial difference between our proposed method and others, the section discussing the differences between the definitions of probability now reads:

> The main advantage of our method is the natural interpretability of these credible regions where an $X\%$ credible region indeed captures the region of parameter space where we expect with $X\%$ probability the actual value of the parameter lies given our statistical model, our prior information, and the observed experimental data. Bayesian methods avoid common misconceptions associated with the construction of frequentists confidence intervals[4].

2. There is no description of the detailed simulation procedure in the main text or in SI. This would make reproducing the results difficult. The reader must also understand the factors that have been taken into consideration during the simulation in order to determine (i) whether the results presented are related to the reader's own experimental environment or not, and (ii) whether the performance assessment based on the simulation is just expected since the simulation entails exactly the same type of noise as those considered in the inference model. I understand from the manuscript that (ii) is exactly the case. I am not saying this approach is wrong, but it certainly needs clarification. Even better, sometimes the simulation may include some additional noise or perturbation that the inference model is unable to account for, so that one can determine whether the inference is robust against those specific additional noises or perturbations.

We thank the reviewer for highlighting this relevant lack of information. We have added all the relevant parameters to Section S1.4 in the supplementary materials describing the logistic growth simulations for each of the figures in the main text, along with a link to the exact file used to generate the synthetic data. Furthermore, as we are fully committed to reproducibility in science, every result in this work can be exactly reproduced by any user since each simulation, analysis, and figure generation script can be found on the GitHub repository for this paper.

Furthermore, based on the reviewer's concern, we added a supplementary material section (Section S1.5) describing the performance of our method on non-logistic growth simulations. Specifically, we reproduced simulations like those presented in Li et al. [3], where the noise in growth, cell sampling, PCR amplification, and sequencing are explicitly modeled. Our method is still able to account for those types of noise sources. Figure S6 in the text (Figure 6 in this document) shows the performance of our model with this more detailed simulation. One interesting observation, shown in Figure 6(E), is that our inferred relative fitness values and the values reported by the code provided in[3] are highly correlated; however, the axis scales vary widely. We conclude that Li et al. [3] fits a different model where they assume a specific functional form for the cellular growth dynamics—Malthusian fitness, i.e., exponential growth—while our model is agnostic to these dynamics and only assumes a functional form for the changes in the relative frequency rather than the absolute number of cells within a culture.

3. There is a disconnect between the results and the actual experimental data. In all tests, inferences are drawn based on simulated data. I understand that it gives the authors the ground truth fitness. Nevertheless, every simulation is based on some simplification or assumption that may not be valid in reality. It is difficult to determine the relevance of the method without actually relating the model to real data.

We thank the reviewer for bringing up this shortcoming of our manuscript. Before directly

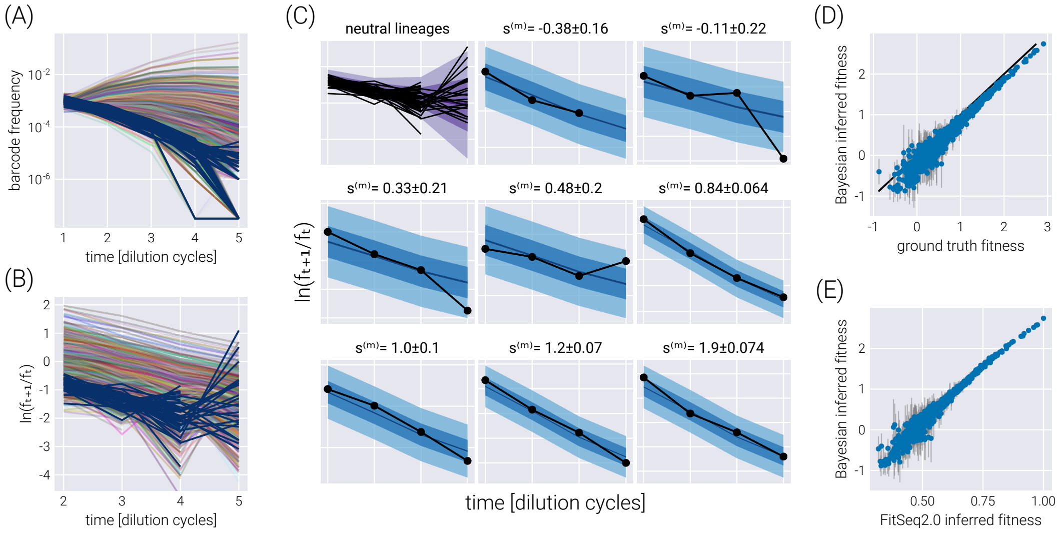

Figure 6. FitSeq2.0 simulation comparison.(A) Frequency trajectories that represent the raw data going into the inference. (B) Log frequency ratio between two adjacent time points used by the inference pipeline. Darker lines represent the neutral barcodes. These transformed data are much more noisy than the seemingly smooth frequency trajectories. (C) Examples of the posterior predictive checks for all neutral lineages (upper left panel) and a subset of representative mutant lineages. Shaded regions represent the 95%, 68%, and 5% credible regions for the data. The reported errors above the plot represent the 68% credible region on the mutant relative fitness marginal distribution. (D) Comparison between the ground truth fitness value from the logistic-growth simulation and the inferred fitness value. Gray error bars represent the 68% posterior credible region for the relative fitness values. (E) Comparison between fitness inferences as obtained using `FitSeq2.0` from Li et al. [3] and our Bayesian method. Vertical gray error bars represent the 68% posterior credible region for the relative fitness values.

addressing the following points brought up by the reviewer, we want to mention that our Supplementary Material now includes a re-analysis of part of the data in Kinsler et al. [1]. In that section, we show that our inference method is able to capture real data based on the experimental design for which `BarBay.jl` was written. Furthermore, the proposed hierarchical model for biological replicates presented in the main text can achieve equivalent levels of agreement with the data while increasing the replicate-to-replicate correlation in the inferred fitness values. We direct the reader to the summary of the results under comment 3.2 below.

> I would like to provide two more specific comments in this regard.
> 3.1. The authors seem to assume some specific composition of the competing population (e.g. most strains are not barcoded), as well as some specific distribution of mutational fitness effects (s) (most barcodes are slightly more adaptive than wild-type). In my opinion, both assumptions are frequently violated. For example, in https://www.science.org/doi/10.1126/science.aae0568, https://www.nature.com/articles/nature17995 and https://academic.oup.com/mbe/article/39/5/msac086/ all genotypes are barcoded. In the commonly used "deep mutational scanning" assays, where only proximal mutations are tested, most mutants (containing only a few simple mutations) have fitness very similar to that of the wild-type. Can the model accurately estimate their fitness? What is the significance of their differences with wild-type?

We thank the reviewer for this thoughtful comment. We agree that the population composition and experimental design assumed in our work do not directly address the structure of other pieces of work, such as those in deep mutational scanning experiments. Specifically, the use of an ancestor strain as a reference against which the relative fitness is computed facilitates the computation

In our view, there are three main reasons for the experimental design we and others in the community (see[5,6]) implement for these competition assays:

- A theoretical reason where the exponential change in frequencies in our fitness model depends on the fraction of each genotype being significantly smaller than one. This is because the functional form of the frequency change between time points (Eq. 3 in the main text)
$$f_{t+1}^{(b)} = f_t^{(b)} \mathrm{e}^{(s^{(b)} - \bar{s}_t)\tau}, \tag{3}$$
necessarily assumes that the frequency of barcode b, $f_t^{(b)} \ll 1$. One can think of Equation 3 as an approximate solution to the classic result in population genetics for the change in frequency of an allele due to selection only, which takes the form
$$\frac{df^{(b)}}{dt} = s^{(b)} f^{(b)} (1 - f^{(b)}). \tag{4}$$
For the case where $f^{(b)} \ll 1$, the term $1 - f^{(b)} \approx 1$, giving an exponential form for the solution.

- A biological reason where frequency-dependent selection effects are minimized. In other words, since the majority of the culture is dominated by a single genotype—the neutral reference genotype—any frequency-dependent fitness effects for the barcodes of interest should be minimized as they do not reach significant fractions of the culture throughout the measurement.
- A chemical reason where the environment experienced by all genotypes is effectively dictated by the growth cycle of the dominant genotype.

We agree with the reviewer that extending the scope of our analysis pipeline to other experimental setups should be a priority for immediate future work. One of the challenges to address is the computation of the population mean fitness without the reference strain. Other approaches, such as FitSeq2.0[3], use optimization algorithms that do not directly translate to our Bayesian framework, so more work must be done to cover other experimental designs fully.

> 3.2. There should be some analysis based on actual experimental data (such as those in the three papers mentioned above). A straightforward test would be to determine whether the Bayesian-inferred fitness shows better between-replicate-correlations than the more naively estimated fitness reported in those papers. I am certain that the author can come up with more analyses relating the actual experimental data to the accuracy of their methodology.

We thank the reviewer for this analysis suggestion to make our contribution more solid and complete. As mentioned before, we added a new section in the supplementary material where we re-analyze data from Kinsler et al. [1]. Briefly, we chose four out of the ≈ 40 environments in the dataset to analyze with our Bayesian algorithm. We applied both a single-dataset analysis and a hierarchical model for multiple biological replicates for these four environments. The example posterior predictive checks show that both models are able to capture the variability within the data, as shown in Figure 7 and Figure 8. However, Figure 9 shows how a hierarchical model for biological replicates significantly increases the replicate-to-replicate correlation.

The supplemental material section includes further analysis of this data. In order to keep our response in this document brief and concise, we invite the reviewer to check the full analysis.

Minor

> 1. The schematic diagram in Figure 1 could use a lot more details, especially regarding the source of uncertainties (at least those have been considered in the model)

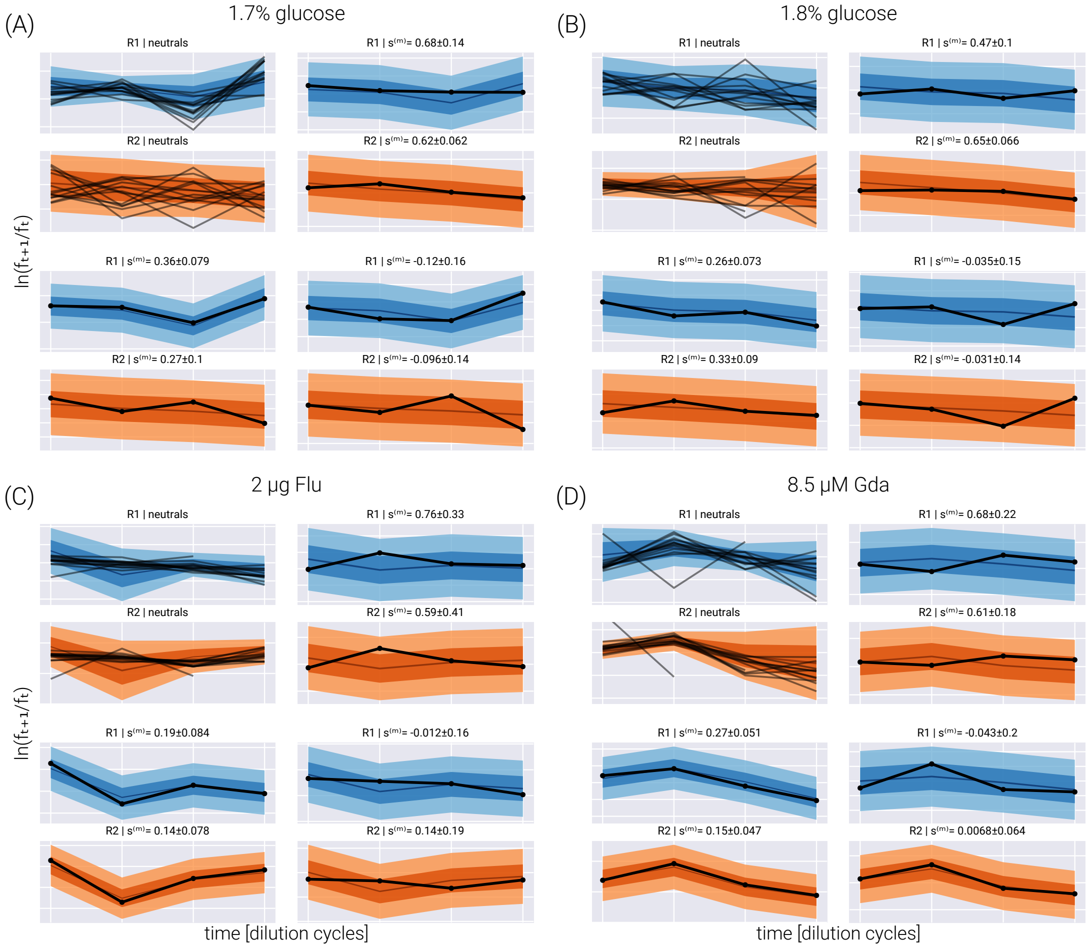

Figure 7. Posterior predictive checks for Kinsler et al., 2020 independent replicates. Examples of the posterior predictive checks for all neutral lineages (upper left panels) and a subset of representative mutant lineages for four environments from[1]. Shaded regions from light to dark represent the 95%, 68%, and 5% credible regions. Listed fitness values for mutant lineages represent the 68% credible region.

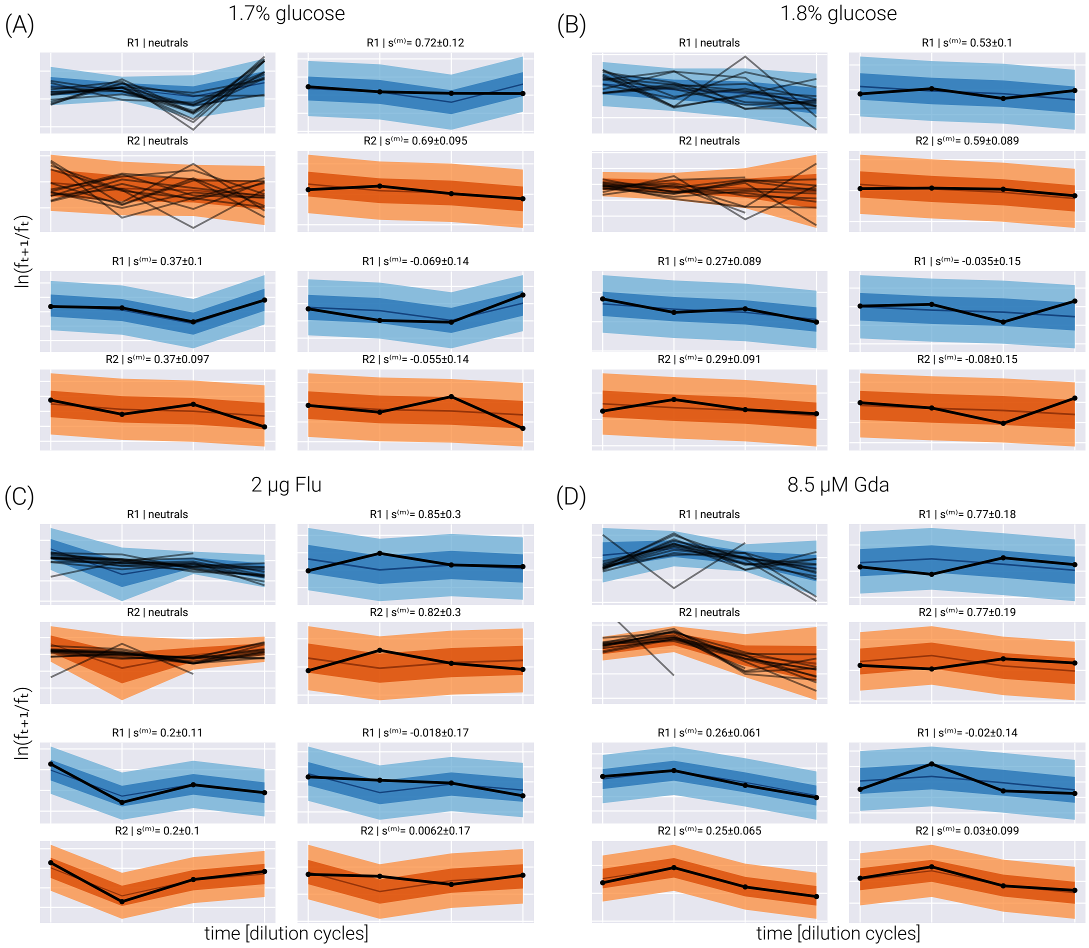

Figure 8. Posterior predictive checks for Kinsler et al., 2020 hierarchical model. Examples of the posterior predictive checks for all neutral lineages (upper left panels) and a subset of representative mutant lineages for four environments from[1]. Shaded regions from light to dark represent the 95%, 68%, and 5% credible regions. Listed fitness values for mutant lineages represent the 68% credible region.

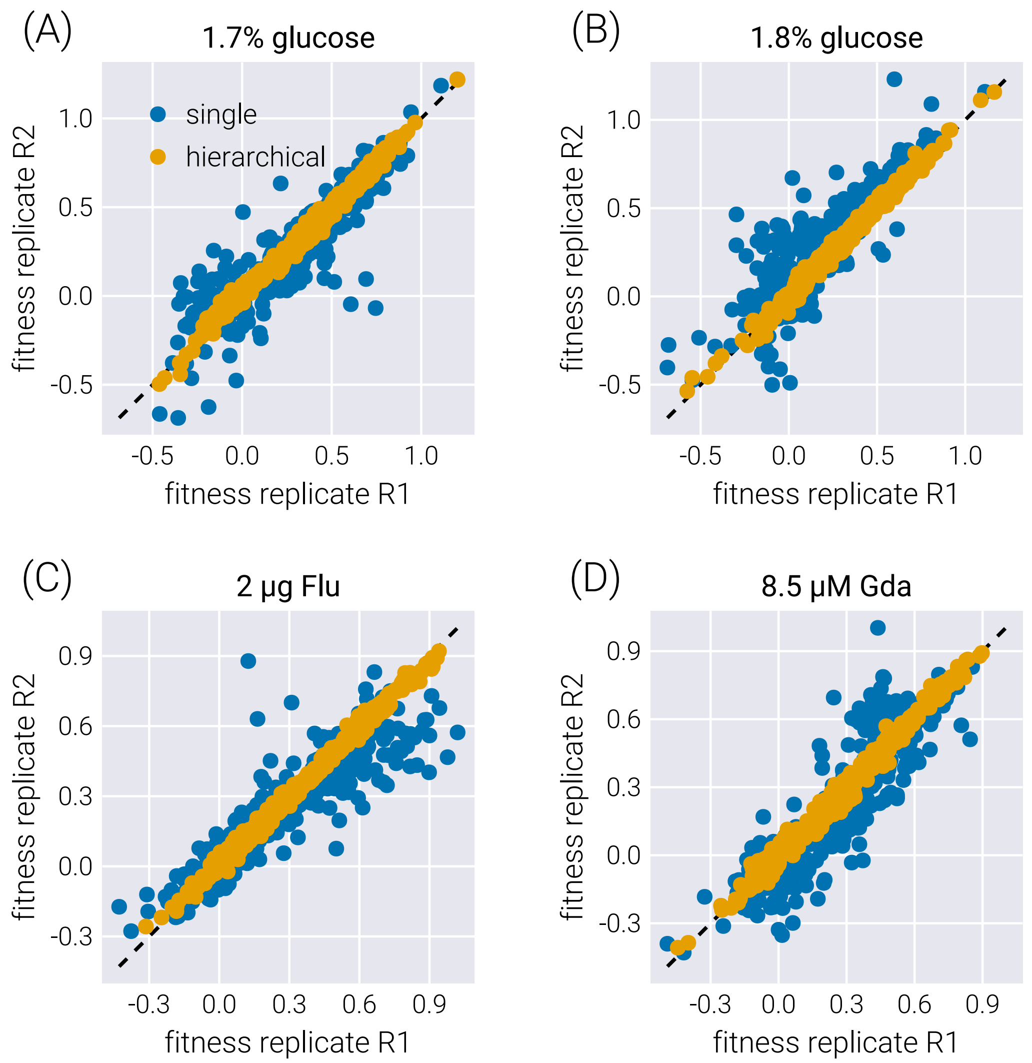

Figure 9. Replicate-to-replicate correlation for both methods. Comparison of **mean** inferred fitness values between experimental replicates when fitting a single-dataset model (blue points) vs. a hierarchical model on the biological replicates (orange points)

We thank the reviewer for this excellent suggestion. We have modified Fig. 1 in the main text, adding a new sub-figure. Here is the new version of the figure:

Furthermore, we added the following line to the main text:

> Figure 1(C) (Figure 10 in this document) shows schematically the sources of uncertainty accounted for by our model. The first three sources—population composition, barcode read counts, and barcode frequencies—all contribute to uncertainty source 1. Uncertainty source 2 and 3 are depicted by the last two sources, respectively. To account for all these sources of uncertainty in a principled way, in the next section, we develop a Bayesian inference pipeline.

2. Figure2D and E. This is related to major comment 2. Please explain how the "ground truth" is chosen/defined and used in the simulation. Also, why "68%" is used ? The number just seems weird.

We thank the reviewer for this suggestion. As mentioned in our answer to Major Comment 2, we have added the corresponding information on how the ground truth values were randomly sampled in the corresponding supplemental information section. Briefly, the growth rates for the non-neutral lineages were sampled from a truncated normal distribution—truncated not to allow unrealistic relative fitness values—around the neutral lineage growth rate value. The specific values for each simulation used in the main text are now listed in the supplementary section.

As for the selection of the 68%, the number is based on the so-called 68–95–99.7 rule commonly used in statistics. This rule, based on the Gaussian distribution—appropriate for our variational approximation—states that 68% of the probability density is capture within $\pm$ one standard deviation from the mean. Likewise, 95% of the probability density lies within $\pm$ two standard deviations from the mean. We avoided stating the use of $\pm$ one standard deviation from the mean to emphasize that our proposed method does not focus on giving a couple of summary statistics but a full posterior distribution. This point, we believe, is important to the Bayesian nature of the computations we are proposing.

3. Figure6F. It would be more prefessional to connect the two lines reaching y=1.0 early on, to the top right corner of the plot area (ECD = 1 across that range).

We thank the reviewer for this suggestion. The corresponding figure has now been updated based on this comment.

4. Line 310, "This loses the subtle differences due to biotic and abiotic batch effects, effectively halving the data that goes into our inference problem" I don't understand this, please elaborate.

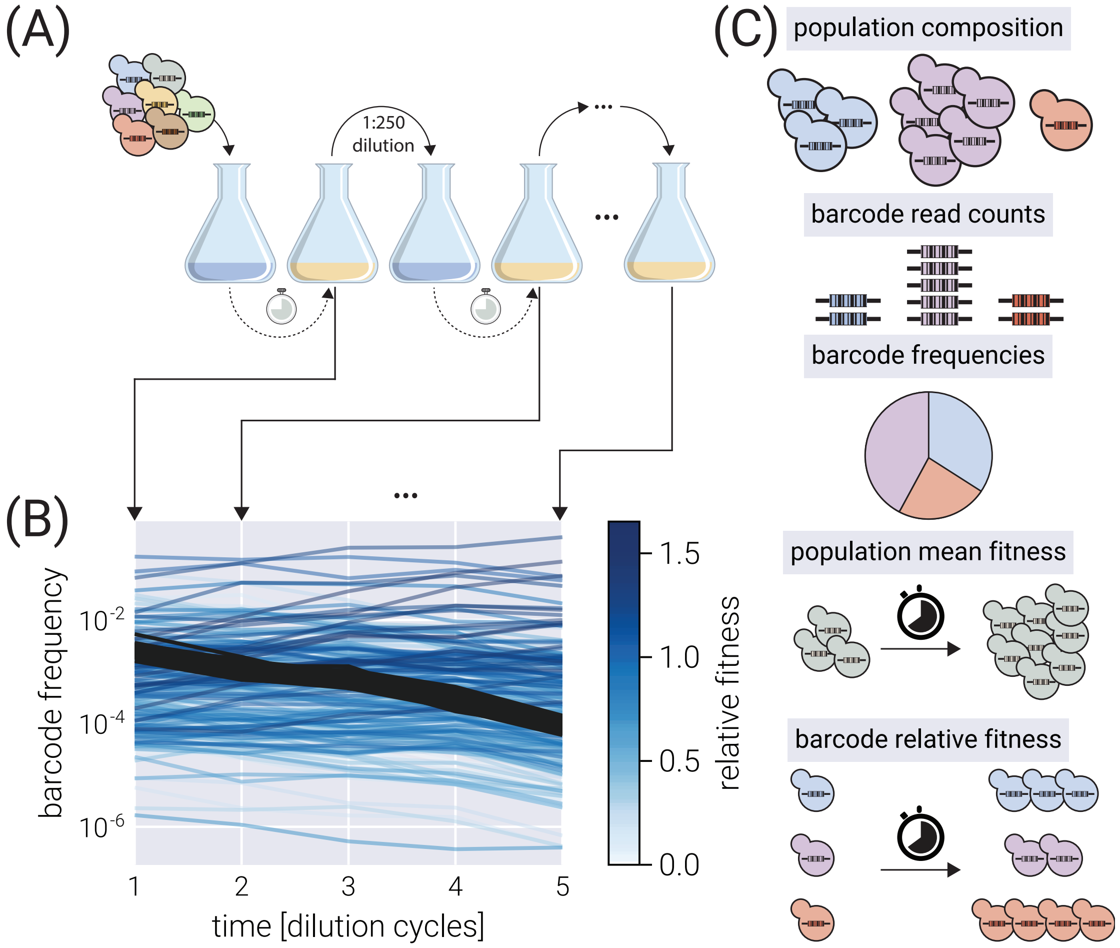

Figure 10. Typical competitive fitness experiment. (A) Schematic of the typical experimental design to determine the competitive fitness of an ensemble of barcoded genotypes. Genotypes are pooled together and grown over multiple growth-dilution cycles. At the end of each cycle, a sample is processed to generate a library for amplicon sequencing. (B) Typical barcode trajectory dataset. From each time point, the relative frequency of each barcode is determined from the total number of reads. Shades of blue represent different relative fitness. Darker gray lines define the typical trajectory of neutral lineages. (C) Sources of uncertainty accounted for by our method. The Bayesian model fit to the data propagates uncertainties from the categories schematically depicted to all parameters in the inference.

We thank the reviewer for highlighting the shortcomings of our statement. We meant to say that given the two extremes of how to handle multiple experimental replicates—analyzing the replicates independently or pooling the data together—combining datasets as a single experiment with deeper sequencing depth effectively removes the information about experiment-to-experiment variability from the data. To exemplify this, let us imagine two experimental replicates sequenced deeply so that the number of reads to barcode fraction conversion was effectively noiseless. Even in this extreme case, when adding more reads would not effectively change the inferred fitness values for each experiment, we would expect the inferred relative fitness values to differ from each experimental replicate. This is because the variability in the inferred fitness values do not only depend on measurement noise but on the intrinsic biological variability and environmental perturbations out of our control—such as small temperature fluctuations. Therefore, pooling the data together smears out that variability. And, unless the number of replicates was large, there is no statistical reason to think the resulting inferred fitness value is significantly closer to the "true" fitness value. On the other hand, hierarchical models explicitly model this experimental variability as each replicate can vary in their local inferred values, sharing information through the hyper-fitness parameter. To clarify this point, we modified that section which now reads as

> On the other hand, we can pool all data together and treat our different experiments as a single measurement with higher coverage. However, by doing so, we lose all information about experiment-to-experiment variability due to intrinsic biological variability and environmental fluctuations—such as small temperature fluctuations—that we cannot control. In this sense, combining the datasets defeats the purpose of performing multiple measurements to account for this variability when extracting the relevant information from the observations.

5. I am unable to see the name of the particular section whenever the author refers to one (for example, line 53, "... See Section ?? for details...", also in line 56, etc.). I have tried two PCs (both using Acrobat on Win11). Are there invisible symbols due to PDF transformation?

We deeply apologize for this inconvenience. This issue with the lack of references to all sections and subsections is now fixed throughout the document.

Reviewer 3

> In Razo Mejia et al, the authors describe a Bayesian framework for the analysis of barcode fitness assays. The authors show how general the framework can be, and test the validity of the inference on synthetic data. I really enjoyed the reading and seeing this type of inference approach finally seeing the light is a blessing to the community. I particularly enjoyed the section dealing with hyperparameters and how it can be used for interesting assays. While it is not the first time barcode fitness inferences have taken a Bayesian spin, it is the first to do so with a true Bayesian perspective on many different parameters. Unfortunately, I am not familiar with Julia (and don't have it installed) and so cannot evaluate the software within the allotted review timeframe as the documentations are fairly lengthy.
> The only major issue I have is that I am unable to judge how much this framework improves on the inference compared to the general approaches the community have undertaken. How poor are the current approaches implemented by the community? Or is it less so about the fitness estimates in the simple case (single environment, assuming replicates are same fitnesses), and more about the fact that the approach can be expanded? Or is it just the credible intervals on fitness values (augmented by priors)? Because my understanding is that almost all the ad hoc approaches taken currently by the community (by the lab of Levy, Petrov, Sherlock, Desai, Dunham, Gresham, or even earlier by the genomic era through the barcoded yeast deletion collection). are very adequate for their purposes. It would have been interesting and borderline necessary to compare at least the simple approaches to this. If it is simply differences between confidence intervals and credible intervals, then it would be ideal if the authors show distinct differences in their output notwithstanding the philosophical differences between them as the differences are usually fairly minimal when priors are not strong.

We thank the reviewer for this excellent comment. The reviewer accurately raises important points about the relevance of our work compared to what preceded our contribution. Indeed, the state-of-the-art methods to infer relative fitness, such as `FitSeq2.0` from Li et al. [3], are adequate for their purposes. Our contribution with the present analysis pipeline is the principled quantification of uncertainty in the reported parameters. In other words, as the author suggests, our main contribution is related to the reported credible regions. For example, although we deeply admire the work presented in[3] that even served as inspiration for our work, in the paper they explicitly mention:

> Finally, Fit-Seq2.0 is still unable to estimate the confidence intervals for each fitness estimate; however, this is something we aim to incorporate into further updates of Fit-Seq2.0.

We see the Bayesian paradigm as the most natural way to quantify uncertainty. Although we agree that the quantitative differences between frequentists and Bayesian error bars can

be minimal for certain models under weakly informative priors; we still think that the transparency of the Bayesian credible region interpretation justifies our method. Furthermore, it is because of this statistical framework that we were able to implement the types of hierarchical models that we present in the later part of the manuscript.

Moreover, based on this and other reviewers' suggestions, we compared our inference pipeline results with those of[3]. We direct the reader to Figure 11 below with the accompanying explanation to see the results of this comparison.

> I have a few minor comments:
>
> 1. There are a few missing references to specific sections (e.g. line 53, line 56, but there are at least dozens throughout).

We deeply apologize for this inconvenience. This issue with the lack of references to all sections and subsections is now fixed throughout the document.

> 2. I'm very familiar with the experimental setup, but I think the readers will not appreciate why the unlabeled reference strain should be at ~90% of the population in these assays as described in lines 69-75. Many barcode assays have not done this in the literature and as far as I know there have been no reported issues. From my recollection, this is done due to frequency-dependent selection on some key lineages, but there is no evidence that this design resolves this (and frequency-dependent selection is not solved by any inference approach).

We thank the reviewer for highlighting this potential blind spot for readers unfamiliar with the experimental setup. In our view, there are three main reasons for the experimental design:

- A theoretical reason where the exponential change in frequencies in our fitness model depends on the fraction of each genotype being significantly smaller than one.
- A biological reason correctly mentioned by the reviewer in which frequency-dependent selection effects are minimized.
- A chemical reason where the environment experienced by all genotypes is effectively dictated by the growth cycle of the dominant genotype.

To reflect these points, we extended the discussion of the design that now reads:

> ... Furthermore, a fraction of labeled genotypes equivalent to the unlabeled reference strain—hereafter defined as *neutral* lineages—are spiked in at a relatively high abundance ($\approx 3 - 5\%$). The rest of the culture is left for the ensemble of genotypes of interest. This experimental design in which the barcodes of interest represent a small fraction of the culture serves three main purposes: First, the model used to infer the relative fitness differences between genotypes is valid in the regime where the genotype frequency is significantly smaller than one (see

Section 2.3). Second, potential problems with frequency-dependent selection are minimized as long as the frequency of each genotype remains small. Third, having a single genotype dominating the culture standardizes the environment experienced by all genotypes. This is because variations in the chemistry of the environment are effectively dictated by the dominating genotype, allowing for reproducible growth-dilution cycles.

Finally, we agree that solving the frequency-dependent selection inference problem would be an important direction to follow. To our knowledge, no piece of work, including this one, has satisfactorily addressed the problem. In the discussion, we mention this is an interesting direction for future work.

3. Is the simulation really sufficient for this work? Without going overboard and testing every single scenarios, it seems overly simple for the power of the proposed approach. I guess I see drift being implemented through the Poisson noise but is the Gaussian noise really adequate for the read frequency if one does not sequence to extremely high coverage? I'm also wondering if systematic noise that all play a role influence the framework at all: such as prolonged lag phase leading to uncertainty on the generations per transfer, exponential jackpotting issues during sequencing that may be poorly estimated from the neutral lineages, day to day variations etc. The main issue is that I feel like all the older approaches can adequately infer fitness for the simulation that was performed.

We thank the reviewer for raising these concerns about the potential shortcomings of our simulations. To extend the scope of our work based on this concern, we added a new supplementary section in which we implement detailed simulations such as those from Li et al. [3]. In these simulations, we explicitly include sources of noise from growth and dilution, cell sampling for library preparation, PCR amplification, and sequencing. Figure 11 shows the performance of our inference pipeline with this more detailed simulation. One interesting observation, shown in Figure 11(E), is that our inferred relative fitness values and the values reported by the code provided in[3] are highly correlated; however, the axis scales vary widely. We conclude that Li et al. [3] fits a different model where they assume a specific functional form for the cellular growth dynamics—Malthusian fitness, i.e., exponential growth—while our model is agnostic to these dynamics and only assumes a functional form for the changes in the relative frequency rather than the absolute number of cells within a culture.

We agree that previous methods can adequately infer relative fitness from the type of simulations we implemented. Moreover, to our knowledge, none of the existing methods, including ours, can explicitly account for the real concerns that the reviewer highlights. However, the primary goal of our method does not lie in the ability to report a single estimate of the fitness but to report a full Bayesian posterior probability. In other words, what distinguishes our method is the principled way of handling, propagating, and reporting the uncertainty in the quantities that researchers use down the line for further analysis. However, we hope that the comparison with this more detailed simulation and actual experimental data—analyzed and

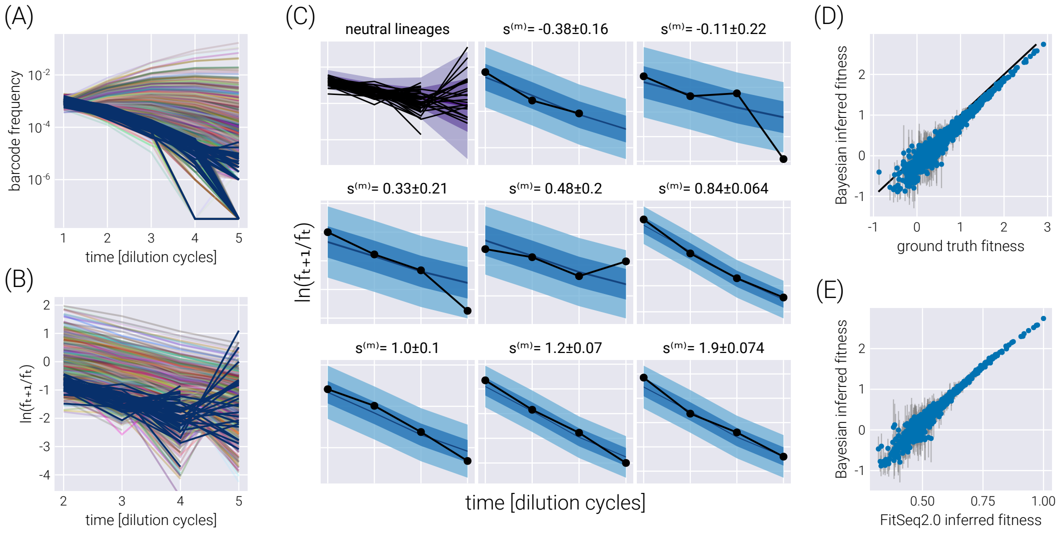

Figure 11. FitSeq2.0 simulation comparison.(A) Frequency trajectories that represent the raw data going into the inference. (B) Log frequency ratio between two adjacent time points used by the inference pipeline. Darker lines represent the neutral barcodes. These transformed data are much more noisy than the seemingly smooth frequency trajectories. (C) Examples of the posterior predictive checks for all neutral lineages (upper left panel) and a subset of representative mutant lineages. Shaded regions represent the 95%, 68%, and 5% credible regions for the data. The reported errors above the plot represent the 68% credible region on the mutant relative fitness marginal distribution. (D) Comparison between the ground truth fitness value from the logistic-growth simulation and the inferred fitness value. Gray error bars represent the 68% posterior credible region for the relative fitness values. (E) Comparison between fitness inferences as obtained using `FitSeq2.0` from Li et al. [3] and our Bayesian method. Vertical gray error bars represent the 68% posterior credible region for the relative fitness values.

described in a new supplementary section—demonstrates the applicability of our inference pipeline.

> 4. I think the aside debating the fundamental differences between confidence intervals and credible intervals is a bit misplaced. Yet if the authors want to keep this in, then perhaps the frequentist confidence intervals should be made more precise by emphasizing the repeated construction of CIs: "frequentist CIs represent the rangeS (emphasis on plural) of values where X% of ranges ..." since upon repeating the experiments the confidence intervals would differ. As is written (in singular 'range'), it may imply a fixed range of values between repetitions (vs a fixed construction method) and the 'repetition' containing the true population parameter would not really make sense.

We appreciate this constructive criticism from the reviewer. Given this feedback and that of the other reviewers, we have decided to significantly shorten the discussion, focusing on how to interpret the uncertainty computed by our method. The section where that discussion takes place now reads.

> The main advantage of our method is the natural interpretability of these credible regions where an $X\%$ credible region indeed captures the region of parameter space where we expect with $X\%$ probability the actual value of the parameter lies given our statistical model, our prior information, and the observed experimental data. Bayesian methods avoid common misconceptions associated with the construction of frequentists confidence intervals[4].

References

[1]G. Kinsler, K. Geiler-Samerotte, and D. A. Petrov, "Fitness variation across subtle environmental perturbations reveals local modularity and global pleiotropy of adaptation", eLife **9**, 1–52 (2020).

[2]Y. Li, S. Venkataram, A. Agarwala, B. Dunn, D. A. Petrov, G. Sherlock, and D. S. Fisher, "Hidden Complexity of Yeast Adaptation under Simple Evolutionary Conditions", Current Biology **28**, 515–525.e6 (2018).

[3]F. Li, J. Tarkington, and G. Sherlock, "Fit-Seq2.0: An Improved Software for High-Throughput Fitness Measurements Using Pooled Competition Assays", Journal of Molecular Evolution, 10.1007/s00239-023-10098-0 (2023).

[4]R. D. Morey, R. Hoekstra, J. N. Rouder, M. D. Lee, and E.-J. Wagenmakers, "The fallacy of placing confidence in confidence intervals", Psychonomic Bulletin & Review **23**, 103–123 (2016).

[5]J. A. Ascensao, K. M. Wetmore, B. H. Good, A. P. Arkin, and O. Hallatschek, "Quantifying the local adaptive landscape of a nascent bacterial community", Nature Communications **14**, 248 (2023).

[6]M. S. Johnson et al., "Phenotypic and molecular evolution across 10,000 generations in laboratory budding yeast populations", eLife **10**, 1–28 (2021).
